# Present and future of smart functional materials as actuators in microfluidic devices

**DOI:** 10.1039/d5lc00259a

**Published:** 2025-07-02

**Authors:** Sepideh Izaddoust, Isabel Poves-Ruiz, Enrique Azuaje Hualde, Daniel Patko, Larisa Florea, Colm Delaney, Lourdes Basabe-Desmonts, Fernando Benito-Lopez

**Affiliations:** a Microfluidics Cluster UPV/EHU, Analytical Microsystems & Materials for Lab-on-a-Chip Group, Analytical Chemistry Department, University of the Basque Country UPV/EHU Leioa Spain lourdes.basabe@ehu.eus fernando.benito@ehu.eus; b Microfluidics Cluster UPV/EHU, BIOMICs microfluidics Group, Lascaray Research Centre, University of the Basque Country UPV/EHU Vitoria-Gasteiz Spain lourdes.basabe@ehu.eus; c School of Chemistry & AMBER, The SFI Research Centre for Advanced Materials and BioEngineering Research, Trinity College Dublin Dublin Ireland; d IKERBASQUE, Basque Foundation for Science Bilbao Spain

## Abstract

The role of actuators in microfluidic systems is fundamental for accurate measurements and analyses, as they enable precise control over fluid flow by converting various forms of energy—including electrical, thermal, piezoelectric, and electromagnetic—into mechanical motion. The integration of actuators within microfluidic devices facilitates system miniaturization, allowing complex fluidic operations at the microscale. Actuators are essential components in micropumps, micromixers, microvalves, and other fluidic control elements, ensuring accurate handling of very small quantities of liquids. However, the selection of the material type for the actuator is highly dependent on the specific application, as well as on the material composition and structural configuration of the microfluidic device in which it will be integrated. Actuators can feature either moving or static components, and the use of hybrid materials allows for the development of innovative actuation mechanisms. Given the vast range of possible actuator-material combinations, selecting an appropriate actuation strategy is critical for optimal device performance. This review presents recent advancements in microfluidic actuation, with a particular emphasis on material innovations. It explores emerging actuator materials integrated within microfluidic channels, their fabrication and integration methods, activation mechanisms, and functional applications. Additionally, the review provides a comprehensive outlook on promising materials for future microfluidic actuator development.

## Abstract 2

This review covers advances in lab-on-a-chip technology, focusing on smart material actuators, their integration and activation mechanisms, and future prospects with novel materials for next-generation of microfluidic devices.

## Graphical abstract

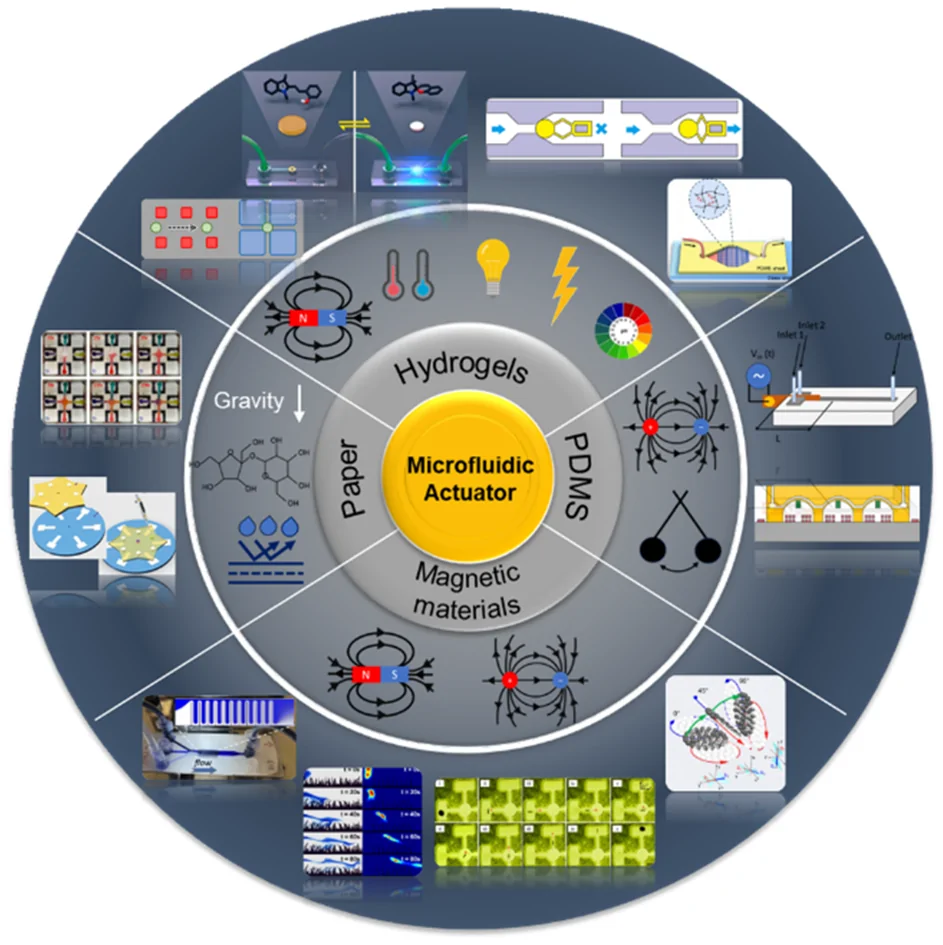

## 1. Introduction

The importance of actuators as fundamental components in microfluidics has long been recognized. The ability to enable precise control over fluid manipulation at the microscale is the key factor in the development of actuators. Since the early stages of microfluidic technologies, actuators have played a crucial role in various applications, including fluid pumping, valving, and mixing. Traditional microfluidic platforms typically were dependent on external pumps, valves, and pressure sources, adding complexity to the system while hindering portability, scalability, and user accessibility. Recent advancements in integrated actuation technologies have enabled on-chip fluid manipulation, mixing, and valving, thereby eliminating the need for bulky external components. These embedded actuators have been shown to enhance automation, minimize reagent consumption, simplify device operation, and improve overall system efficiency. Consequently, they significantly contribute to the development of more reliable, scalable, and commercially viable point-of-care diagnostics and analytical platforms, essential for numerous fields, ranging from chemical analysis to biomedical diagnostics.^1,2^

Conventionally, passive actuators, defined as those that do not require external driving forces to perform their function, have been used over the years.^3^ These actuators could be categorized based on whether they operate through structural design, surface functionalization to add new properties, or the inherent properties of the materials themselves, enabling intriguing “self-powered” fluid manipulation. Different materials have been used for passive actuators such as rigid polymers like poly(methylmethacrylate) (PMMA),^4^ soft polymers like polydimethylsiloxane (PDMS),^5^ inorganics such as glass^6^ or silicon (Si),^7^ paper,^8^ and their combination.

In the context of actuators based the design, rigid polymers such as polycarbonates (PC), PMMA, polyethylene (PE), polypropylene (PP), polystyrene (PS) as well as glass and silica have been widely used in microfluidic devices. High rigidity, stability, and suitability for various manufacturing processes, have made these materials ideal for use as passive mixers^9,10^ to enhance the interaction between fluids by controlling the flow within the microchannels. Employing various geometrical designs of microchannels such as T-shaped,^11^ grooved^12^ and/or herringbone,^13^ increases the contact area between the fluids by inducing a turbulent or chaotic flow.^14,15^ In addition to rigid polymers, glass and silica have been widely used for fabrication of micromixers. Channon *et al.*^16^ designed a self-pumping asymmetric staggered herringbone mixer from laser-ablated glass and tape, achieving rapid flow and mixing without external forces or pumps. In another inventive design, a 3D micromixer made from borosilicate glass and a silicon substrate was employed, using Baker's transformation concept.^17^ These examples demonstrate diverse approaches to develop advanced microfluidic mixers, incorporating materials such as glass and silicon substrates, along with innovative manufacturing techniques such as ultrafast laser micromachining^18^ and femtosecond laser-induced wet etching.^19^ This emphasizes the significance of geometric design and material selection in achieving efficient fluid mixing within microfluidic systems, ultimately advancing lab-on-a-chip (LoC) technologies.

The surface of rigid polymers (*e.g.* poly PMMA, cyclic olefin polymer (COP) and copolymer (COC), PC, poly(-ethylene terephthalate) (PET), PS, poly (ethylene glycol) (PEG), polylactic acid (PLA), acrylonitrile butadiene styrene (ABS), or polyester) and glass can be functionalized in order to modify the surface properties and enhance their performance in biological and chemical environments. This modification allows for control of material wettability or biomolecule immobilization. By adjusting the hydrophobicity or hydrophilicity of the surface, the behaviour of fluids within microchannels can be controlled, optimizing fluid handling and flow dynamics.^20^ As an example, PMMA was functionalized with polyvinyl alcohol (PVA) to develop a capillary-driven flow device for simultaneous multi-sample analysis. The PVA coating transforms the PMMA surface from hydrophobic to hydrophilic, facilitating the drawing of fluid samples into the microchannels.^21^ Indeed, this functionalization approach offers versatility in selecting materials and modifying their properties to suit specific application requirements. It provides a means to tailor the interaction of fluids with the microchannel surfaces, thereby improving the efficiency and effectiveness of microfluidic systems in various analytical and diagnostic operations. Moreover, the surface of rigid polymers can be functionalized by physical attachment, amination and carboxylation in order to improve chemical immobilization susceptible, for instance, for biological applications. These polymers have been commonly used in microfluidic devices due to their biocompatibility, optical transparency, and mechanical strength. For instance, in recent work,^22^ the surface of a PMMA/paper hybrid channel was functionalized by immobilization of proteins on the surface, enabling binding with the target antibody.

Since the inception of microfluidic technologies, porous materials like papers and filter papers have been widely used. Owed to their biocompatibility, cost-effectiveness, and rapid prototyping capabilities, they have found numerous applications ranging from point-of-care detection^23^ to ELISA tests^24^ and even exudate detection of living plant roots.^25^ The inherent porous property of paper facilitates a lateral flow of liquids through the material. This feature makes paper an ideal material to be used as passive micropumps, conventionally the most common type of actuators which do not require external power.^26,27^ The pumping mechanism of paper is based on the capillary forces which typically operate by controlling the flow rate during the filling process, making them useful when precise flow rates without continuous regulation are needed.^28,29^ Moreover, by harnessing the natural wicking properties of paper, it can be effectively utilized as valves in various applications. This innovative use of paper as a valve leverages its ability to absorb and transport liquids, by capillary action, as demonstrated by Hu *et al.*^22^ in their study. In addition, paper can be seamlessly integrated into PMMA channels to enhance their functionality. In these channels, the mechanical pressure applied to the paper can be used to regulate the flow of fluids and to control fluid dynamics.^30^ The combination of the wicking properties of paper and its mechanical responsiveness within PMMA systems offers new possibilities for the design of efficient and cost-effective fluid control mechanisms.

Another material which has a long history in microfluidics technology is PDMS.^31–33^ The advantageous properties of PDMS, which include ease of fabrication and the capacity to precisely replicate the surfaces of molds, render this polymer an ideal material for the fabrication of passive microfluidic components, including channels,^34^ mixers,^35,36^ and actuators.^37^ Furthermore, PDMS can be used to create hybrid materials, thereby extending its application range beyond channel fabrication to microfluidic actuator.^33,38,39^ Electrodes and valves can be readily integrated into a PDMS system,^40^ and can be combined with graphene,^41^ electric coils,^42^ and chromophores.^43^ PDMS has been extensively used for pressure-difference pumps, particularly in the degas-driven flow method. Indeed, effective flow regulation requires materials that have high air solubility like PDMS affording the absorption and release of air under varying environmental pressures, creating vacuum or high pressure inside the channel, which drives fluid motion.^44,45^ PDMS facilitates gas diffusion by creating free volumes within the material. When degassed, PDMS generates a vacuum and reabsorbs air upon exposure to atmospheric pressure, creating negative pressure that drive fluid flow. This process is influenced by microchannel geometry, material permeability, and the balance between gas diffusion and liquid movement.^46–48^ Shen *et al.*^49^ used this technique to develop a microcasting method for patterned cell culture systems, enabling controlled cell adhesion.

The other category of materials with numerous applications in LoC include natural and synthetic hydrogels. These materials are known for their high-water content, flexibility, and ability to mimic natural tissue. The hydrophilic three-dimensional networks of hydrogels, capable of absorbing and retaining significant amounts of water, solvents or biological fluids has made them ideal as passive capillary pumps in pumpless microfluid devices.^50^ Unlike paper, this phenomenon occurs because the weak bonds in hydrogels break upon absorbing liquids, leading to their swelling.^28^ Although, the capillary pressure in hydrogels is lower compared to rigid porous material pumps, and may decrease further when pumping fluids, they still offer superior mass or volume efficiency.^51^ Hydrogels possess several important properties, including, biocompatibility, mechanical strength, biodegradability, swell ability, and sensitivity to external stimuli.^52^ These properties can be adjusted by altering the ratios of hydrophilic to hydrophobic components, the concentrations of initiators, composition of polymers, and the reaction conditions.^53,54^ This makes hydrogels ideal to be either passively or actively used as inert matrices to host a board range of responsive and active materials such as metal nanoparticles^55,56^ biomolecules,^57–62^ living cells^63–66^ as well as ions.^67,68^ They can also be designed to respond to specific stimuli. Stimuli-sensitive, also called, smart hydrogels have been synthesized by adjusting their composition, becoming responsive to physical, chemical and biochemical environmental triggers. Based on the type of crosslink (chemical, physical and/or dual-network) the response of smart hydrogel leads to its volumetric or structural changes.^69^ Based on the composition, smart hydrogels can respond to temperature,^70,71^ electric fields,^72^ light,^73^ pressure,^74^ pH,^75^ ionic strength,^76^ and certain biological molecules.^77^ This feature enables hydrogels to be applied as smart actuators^69,78^ like valves,^79,80^ grippers,^81^ microcages,^82^ micropumps,^83,84^ and membranes.^85^ They are also used in drug delivery,^86,87^ drug release and tissue engineering,^88^ encapsulation,^89,90^ and cell culture,^91^ which demonstrate the importance of these materials in LoC technology.

In addition to the aforementioned materials, magnetic materials have also become a common component of macroscale fluidic systems. These materials form a distinctive class that uses the potential energy stored in magnetic fields to generate mechanical signals, thereby enabling precise and versatile actuation.^92–95^ The family of magnetic materials encompasses a wide range of substances, including magnetic nanoparticles, ferrofluids, and solid ferromagnetic alloys such as stainless steel, as well as composite materials.^96^ Despite their diversity, these materials share a common response to external magnetic fields, whether generated by permanent magnets or electromagnets. Magnetic materials have become a common component of macroscale fluidic systems, where they facilitate the efficient operation of components such as pumps, mixers and valves.^97–99^ In microfluidics, they offer distinct advantages including precise and tuneable actuation, enabling dynamic fluid handling and unlocking novel types of actuation mechanisms previously unattainable with traditional methods.^100–103^ One of the primary benefits of using magnetic materials in microfluidics is the ability to finely control actuation by adjusting the strength and gradient of the applied magnetic field. This level of precision facilitates highly adaptable and responsive fluid manipulation. Furthermore, the integration of magnetic materials into microfluidic devices has resulted in the development of innovative, efficient, and flexible methods of fluid control.^104^ These advancements continue to expand the capabilities of microfluidic systems, making magnetic materials an indispensable tool for modern fluidic applications.

This review provides comprehensive and up-to-date exploration of the diverse materials employed as actuators in microfluidic devices. Given the pivotal role of actuators in regulating microfluidic environments, their importance cannot be overstated. This review aims to guide the selection of suitable materials for specific actuator applications by discussing their fabrication and integration methods, the external stimuli that activate them, and their wide-ranging applications. Additionally, it highlights the advantages of these materials, emphasizing their practicality and ease of implementation. Beyond established materials, this review introduces emerging alternatives that represent the next generation of microfluidic actuators, with the potential to significantly enhance system performance and functionality. By focusing on these advancements, this work underscores the transformative potential of novel materials in LoC technology, paving the way for more efficient, precise, and versatile microfluidic devices.

## 2. Functional materials as actuators

### 2.1 Paper and cellulose based actuators

Paper, as a material, has emerged as a highly suitable platform for microfluidic devices due to its intrinsic properties and structural versatility.^25,105–107^ Its natural wicking ability enables passive fluid transport through capillary action, eliminating the need for external pumps or power sources. Composed primarily of cellulose fibres, paper features a porous structure that can be chemically modified or physically patterned to regulate fluid flow and enhance functionality.^107–109^ Additionally, its affordability, lightweight nature, and availability in various forms make it a practical choice for low-cost and scalable device fabrication. The ease of processing through techniques such as printing, wax-patterning, and lamination further expands its applicability in microfluidics.^110–112^ Beyond its mechanical and chemical adaptability, paper's compatibility with biological and chemical assays has driven its widespread use in applications such as point-of-care diagnostics, environmental monitoring, and biomedical research. Its biodegradability and disposability also contribute to its appeal, particularly in resource-limited settings where cost-effective and sustainable solutions are essential.^113,114^

**Fig. 1:**
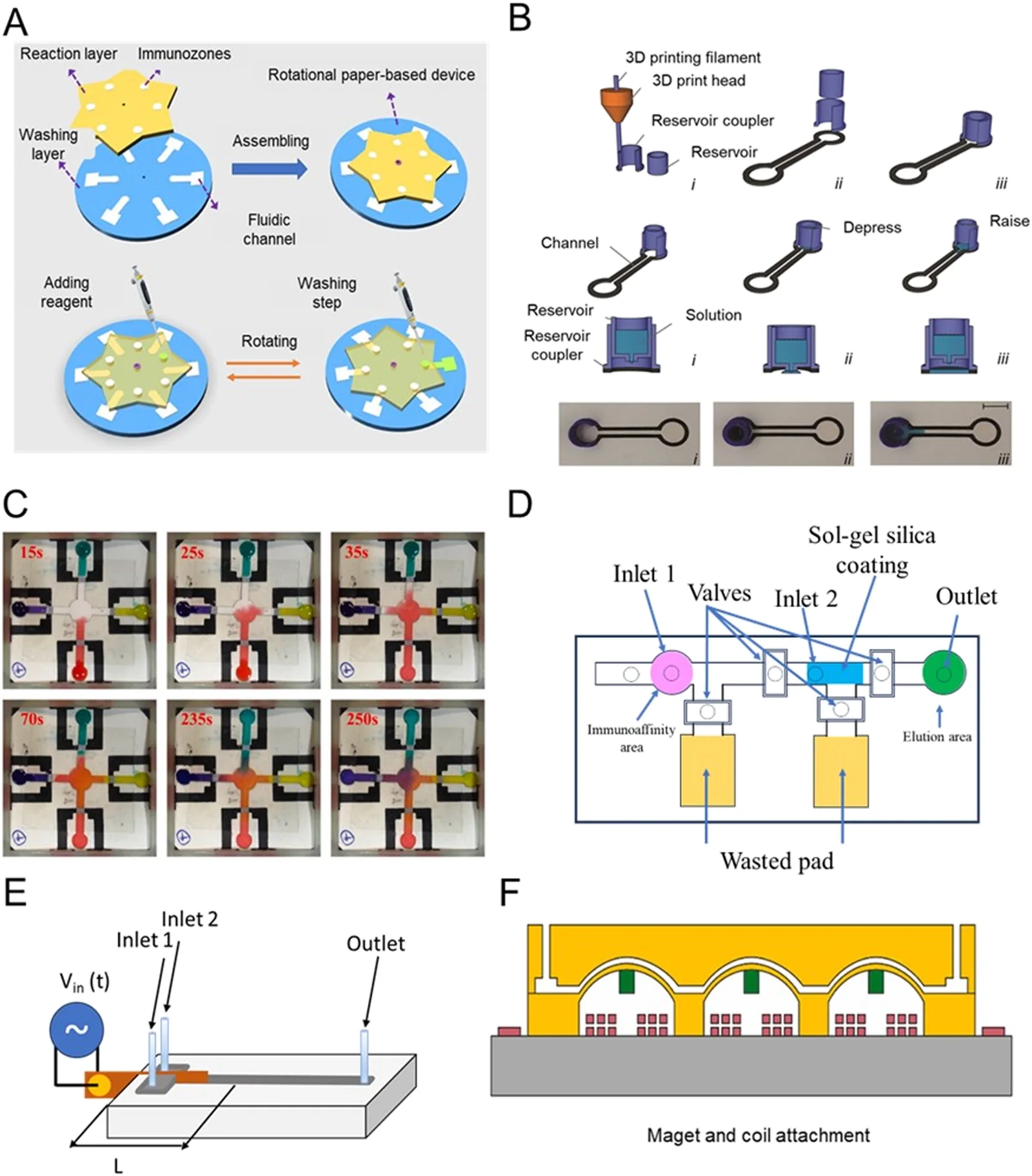
Hybrid PDMS and paper-based actuators in microfluidic devices. (A) Paper-based ELISA test system, where sensors rely on the rotation of paper discs containing assay reagents. The mechanical movement of the discs facilitates the transfer of reagents and samples. Reproduced from Lin *et al.*, with permission from Nature, copyright 2022.^121^ (B) Schematic of finger-actuated hybrid reservoirs and valves demonstrating the suitability of paper combined with 3D printing. (i) Fabrication of components, (ii) mounting onto the hybrid device (iii) slotting reservoir components into the couplers. Reproduced from Zargaryan *et al.* with permission from Nature, copyright 2020.^122^ (C) A micro heater and a wax barrier inserted into a paper microfluidic system, using screen printing techniques. The control of the micro enables precise control of the wax barrier and flow. Reproduced from Atabakhsh *et al.*, with permission from Elsevier, copyright 2024.^125^ (D) Flow in paper can be regulated using screws implemented into a PMMA housing. The pressure of the screw regulates the flow within the paper. Reproduced from Hu *et al.*, with permission from Elsevier, copyright 2023.^22^ (E) A Nafion–Pt electrode-based ionic polymer–metal composite (IPMC) is inserted into a PDMS microfluidic channel to be used as a mixer. Reproduced from Annabestani *et al.* with permission from IEEE, copyright 2020.^40^ (F) Schematic representation of a PDMS device with tiny electromagnets. The magnetic actuators generate peristaltic motion in the channels working as inbuilt pumps. Reproduced from Subandi *et al.*, with permission from Elsevier, copyright 2024.^42^

Beyond paper, several other materials with similar properties have been explored for microfluidic applications, particularly those that leverage capillary-driven fluid transport. Nitrocellulose, widely used in lateral flow assays, offers high porosity and excellent protein-binding capacity, making it an ideal for bioassays and diagnostic applications.^106,109,115^ Textiles, including cotton, silk, and synthetic fibres like nylon, provide flexible and porous substrates that can be patterned or functionalized to guide fluid flow.^112,116^ Cotton threads have been integrated into microfluidic devices as channels for passive fluid transport, offering a simple and scalable alternative to traditional microfabrication techniques.^117,118^ Similarly, nylon membranes provide controlled porosity and chemical resistance, expanding their use in filtration and separation processes.^119,120^ In a study by Lin *et al.*,^121^ a nitrocellulose rotating disc was used as the reaction layer for an ELISA test, paired with a filter paper disc that served as a reservoir for the washing step. To create fluidic channels on the discs, UV-curable polyurethane was screen-printed onto the nitrocellulose surface, effectively defining hydrophobic barriers and guiding fluid flow (see Fig. 1(A)). Paper can also be integrated with 3D printed materials, expanding the design possibilities of microfluidic systems. For instance, at Zargaryan's group,^122^ a 3D printed system was developed which featured a slotted bridge valve that regulated the flow through the paper channels *via* simple mechanical pressure on the paper. Additionally, their design included a 3D printed liquid reservoir connected to a filter paper channel, where simply applying pressure allowed for controlled release of the liquid into the microfluidic network. This integration of paper with additive manufacturing techniques demonstrates a promising approach for creating hybrid microfluidic systems with enhanced control over fluid manipulation (see Fig. 1(B)).

The wicking property of paper can be finely tuned and used to create valves and separate reaction compartments in microfluidics. When paper absorbs water, its weight and persistence length change, leading to structural deformation. As a cantilever, the wicking paper bends downwards, establishing a fluidic connection between previously separated compartments.^123^ Tu *et al.*^124^ developed a complex valve system to regulate flow through paper-based channels. Their design incorporated a sponge attached to a filter paper, which expanded upon water absorption. This expansion displaced a movable arm with a hydrophilic head, bridging hydrophilic channel sections and facilitating fluid transfer. Additionally, they introduced a fructose-based soluble retardant to control flow speed. These strategies demonstrate the potential of paper's inherent mechanical and hydrophilic properties for the development of self-regulating microfluidic devices.

Wax barriers play a crucial role in paper based microfluidic systems, acting as hydrophobic boundaries to direct and control fluid flow. Moreover, it is possible to tune the flow over these wax barriers. Beyond passive containment, the flow over wax barriers can be actively tuned to enable dynamic fluid regulation. In the work of Atabakhsh and colleges^125^ a simple paper-based fluidic system with screen-printed wax micro bridges was developed, combined with screen printed electrodes functioning as thermal actuators (see Fig. 1(C)). By using the electrodes as localized heaters, it was possible to melt and regulate the melting of the wax barriers, modifying the structure of the paper channels and altering the flow conditions on demand. Paper-based systems can also be combined with rigid substrates, such as PMMA, to enhance their mechanical stability and functionality. Hu *et al.*^22^ demonstrated the benefit of using PMMA to securely house fluidic components, such as paper tissue. Additionally, screws were inserted over paper channels, allowing for mechanical actuation of fluid flow (see Fig. 1(D)). This hybrid system enabled the isolation of exosomes and the extraction of miRNA from biological samples, showcasing the potential of paper-PMMA microfluidic platforms for biomedical applications.

### 2.2 PDMS and hybrid PDMS actuators

PDMS is widely used in microfluidics^34^ primarily as passive elements such as channels, or mixer thanks to its geometrical or elastic properties.^35,126^ It has also been employed as an integrated pump, particularly when degassed PDMS is used.^127,128^ Integrating mechanical valves into PDMS-based microfluidic systems is a common practice, especially in 3D structures, where a parallel PDMS layer can be fabricated for pneumatic channels. This layer can apply pressure to seal liquid flow within the liquid channels. Additionally, screws can be inserted into the PDMS to regulate pressure and block flow in the channel.^37,129^

Electrodes can also be used to regulate flow in PDMS-based microfluidic systems. Qaiser's group^130^ integrated a square-wave meander copper-based microheater into a microfluidic chip, achieving a 44% higher output temperature with lower input energy compared to other meander heaters. They placed a PDMS-Expancel hybrid reservoir-channel-outlet system over the microheater, and during thermal expansion, water was pushed out of the reservoir. Additionally, simple sliding wall parts can be inserted into a PDMS systems to create manually controlled valves. In Venzac's work,^131^ a 3D-printed sliding wall was used for valving, pumping, and compartmentalization in a PDMS microfluidic system. They system contained a polymerized polyethylene glycol diacrylate (PEGDA) and an agarose-based hydrogels separating two fluidic compartments. When a constant electric field was applied between the two compartments, DNA migration occurred. However, the PEGDA membrane's pore size (approximately 5 nm) was too small to allow DNA transfer, resulting in DNA concentration in front of the membrane. In contrast, the DNA could easily flow through the agarose gel.

Recent advances have explored the use of PDMS-based hybrid materials as microfluidic actuators, with varying levels of hybridisation. Annabestani *et al.*^40^ developed an ionic polymer–metal composite (IPMC)-based cantilever micromixer into a microfluidic channel. The movement of a Nafion–Pt electrode-based IPMC generated an effective mixing within 15 s (see Fig. 1(E)). In another advancement, electro wetting properties of a PDMS–graphene oxide (GO) hybrid surfaces were modified to achieve precise translocation of water droplets using electrodes. The electrodes, with triangular shapes (1.5 × 1.5 mm) and 60 μm spacing, enabled accurate control of water droplets *via* electrical voltage.^41^

PDMS also allows the incorporation of larger components, as demonstrated by Subandi *et al.*,^42^ who inserted tiny magnets and electromagnetic coils into PDMS to create a peristaltic pump. The system achieved a flow rate of 8 mL min^−1^, with small volume changes generated by the electromagnets (see Fig. 1(F)). Additionally, heating electrodes can be integrated into PDMS-based microfluidic systems due to PDMS's excellent heat resistance. In a multilayer PDMS device, Sesen *et al.*^132^ embedded a heating wire surrounded by oil beneath a PDMS membrane. When the wire was switched on, the generated heat caused the oil to expand, which in turn pushed the PDMS membrane, blocking the flow within the channel above the heater, achieving closure within 2 s. Finally, chromophores can also be incorporated into PDMS to enable light-controlled fluid flow. Angelini and colleagues^43^ used a dispersed red 1 methacrylate material doped into the PDMS, which expanded under a 532 nm light source effectively blocking the fluidic channel and controlling flow.

### 2.3 Hydrogel based actuators

Hydrogel actuators are cutting-edge materials capable of altering their shape or size in response to external stimuli, including temperature, chemicals, pH, light, and electric or magnetic fields.^133^ These actuators are three-dimensional polymer networks that can retain significant amounts of water and other fluids. Smart hydrogel actuators can undergo both reversible and irreversible changes in volume and shape when exposed to specific stimuli. They can be engineered to undergo numerous shape-morphing modes, such as bending, rolling, twisting, folding, origami, walking, and crawling by emulating natural movements. These stimuli-responsive shape transformations have led to the development of various applications, including valves, sensors, and robotics.^69,134,135^ Their biocompatibility makes them ideal for biomedical applications, including drug delivery, tissue engineering, and soft robotics. Additionally, they can be integrated into smart textiles and wearable sensors for health monitoring.^88,136–139^ Hydrogel actuators represent a pioneering advancement in materials science, offering transformative potential across multiple fields due to their exceptional responsiveness and adaptability.

The application of hydrogels as actuators in LoC technology originated two decades ago when Beebe *et al.*^140^ integrated hydrogel valves into microfluidic systems for self-regulated flow control inside the channel. They used direct photopatterning of liquid-phase hydrogels within microchannels, simplifying system construction and assembly, which overcame traditional challenges in microscale integration and improved system functionality. Similarly, a thermo-responsive valve was developed by Wang *et al.*^70^ in order to be used for flow control, distribution of the samples into multiple paths, metering and sealing of a polymer chain reaction (PCR) reactor.

**Fig. 2:**
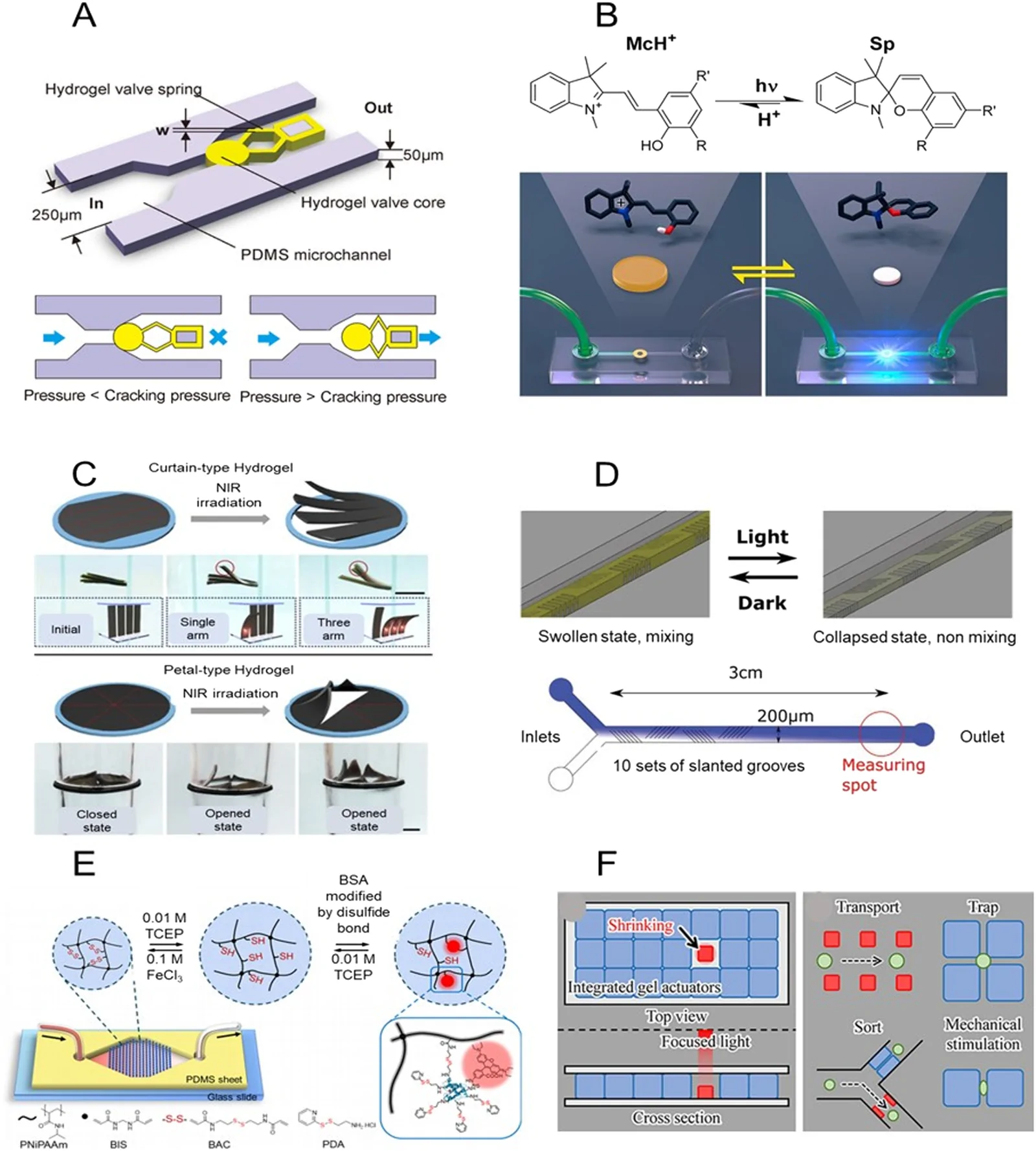
Hydrogel actuators in microfluidic devices. (A) Valve for low-pressure operations. It offers near-zero forward cracking pressure, no reverse leakage, and tuneable fluidic resistance. It shows high repeatability, making it ideal for use in finger-actuated micro-mixers and bio-actuated micro-pumps. This design significantly simplifies flow control in integrated microfluidic systems. Reproduced from Wang *et al.* with permission from Elsevier, copyright 2020.^148^ (B) Light-responsive hydrogel microvalve, which operates through the isomerization of protonated merocyanine (McH^+^) and spiropyran (Sp) forms when exposed to light. This light-induced isomerization causes changes in the hydrogel's size, enabling the microvalve to function effectively within microfluidic systems. Reproduced from Ter Schiphorst *et al.* with permission from ACS Publications, copyright 2015.^155^ (C) Hydrogel valve exhibits significant pressure resistance, facilitating light-controlled solid/liquid transportation and enabling a controllable reaction switch. Produced from Cheng *et al.* with permission from Elsevier, copyright 2019.^156^ (D) Photo-responsive passive micromixers using a light-responsive hydrogel. In the off-state, the hydrogel is swollen and acts as a passive mixer, upon light exposure, it shrinks, resulting in a non-mixing flat state. Reproduced from Ter Schiphorst *et al.* with permission from Wiley, copyright 2018.^158^ (E) Reversible protein capture and release using a redox-responsive hydrogel integrated into a microfluidic chip. Modified BSA is captured and released *via* disulfide bond cleavage and reformation within the hydrogel dots. Reproduced from Jiao *et al.* with permission from MDPI, copyright 2022.^159^ (F) Light-driven gel actuator for single-cell manipulation is illustrated, demonstrating the actuation of a single hydrogel within an array of multiple actuators. Reproduced from Koike *et al.* with permission from Frontiers, copyright 2020.^160^

Harmon and colleagues^141^ developed an innovative actuation system that used a thermo-responsive *N*-isopropylacrylamide (NIPAAM) hydrogel valve integrated with a PDMS membrane. This design separated the microfluidic channel from a reservoir, allowing the actuator to swell and enabling precise flow control for various solutions across a wide range of pH and ionic strengths. In another study, Gieger's group^71^ introduced a versatile, disposable polymer-based microfluidic device with integrated fluidic interconnects for high-pressure operation, lithographically patterned microheaters, and a thermally sensitive hydrogel valve. This valve remains closed at room temperature and opens above 32 °C, allowing fluid flow. The device demonstrated reliable actuation over 100 cycles, with a rapid valve response time of 5 s using on-chip heaters. On the other side, Haefner *et al.*^142^ developed a large-scale integration (mLSI) platform, utilizing up to 172 hydrogel valves per square centimetre in a single microfluidic circuit, controlled using an optoelectrothermic transducer setup for precise manipulation of the microfluidic system. This device enabled parallel execution of thousands of reactions on a single chip, highlighting the potential for advanced micro-electro-mechanical systems (MEMS) applications and complex fluidic operations with high efficiency and reliability. Additionally, innovative microvalves made from pH-responsive hydrogel materials were created within microchannels using liquid phase polymerization. This approach simplified device construction and enabled both sensing and actuation functions. Liu and colleagues^143^ detailed the fabrication process, employing in-channel processing and *in situ* photopolymerization techniques such as the “laminar stream mode” and “mask mode”. They produced several 2-D and 3-D hydrogel-based microvalves, which were tested for response time, pressure drop, and maximum differential pressure capabilities. pH-responsive hydrogels were used to generate the actuation pressure required for both valving and dispensing functions in a microfluidic device. An array of these responsive hydrogels was used in a microdispensing device to deform a flexible membrane above a fluid reservoir chamber. This deformation altered the chamber volume, pushing fluid through when the valve was open. When the valve was closed, the expanding hydrogel array created a storable pressure source for fluid dispensing upon reopening.^144^ Besides pH and thermo-responsive hydrogels, researchers explored environmentally sensitive hydrogel microvalves for microactuation in aqueous media. These microvalves facilitated rapid swelling and deswelling transitions in response to the diffusion of chemical species through a porous membrane, causing a flexible diaphragm to deflect and open or close the valve orifice. The hydrogel, based on phenylboronic acid, showed sensitivity to glucose and pH variations, making it ideal for applications such as drug delivery systems.^145^ Exploiting the swelling property of hydrogels, Kim *et al.*^146^ developed active walls for blocking/diverting flow and delivery pistons for moving objects within microchannels. This was achieved using in-channel liquid-phase photopolymerization (LP3) technology to fabricate reconfigurable components within integrated systems. Leveraging the elastic force of a swollen hydrogel, the same group^147^ used microfluidic tectonics and liquid-phase photopolymerization to create an in-plane bi-polymer check-valve designed for microfluidic flow control and contamination prevention. The valve operated actively through hydrogel swelling and passively as a one-way valve, offering quick restoration of the valve head using elastic forces. Wang's group^148^ applied a photo-curable hydrogel solution containing PEGDA to fabricate a planar micro check valve *via* optofluidic lithography. This valve featured simple fabrication, easy integration with microfluidic devices, and excellent performance in low-pressure operations, including nearly zero forward cracking pressure, no reverse leakage, adjustable fluidic resistance, and good repeatability. They demonstrated the valve's potential in finger-actuated micro-mixers and bio-actuated micro-pumps, thereby simplifying flow control in integrated microfluidic systems (see Fig. 2(A)).

Hydrogel materials function based on their ability to absorb and release water, a process determined by the lower critical solution temperature (LCST) behaviour. Below the LCST, hydrogels absorb water and swell, while above this temperature, release water and shrink. By employing a low energy light source such as light emitting diodes (LEDs), it is possible to convert light to heat and actuate these gels through LCST behaviour, which make them particularly attractive.^149–152^ Moreover, light-responsive hydrogel actuators can be developed by optimizing the molecular design of spiropyran photochromes.^153^ Sugiura and his team^154^ developed an innovative photo-responsive microvalve that operates through localized photopolymerization. They achieved this by integrating a spiropyran-based moiety into the hydrogel and channelling acidic water through it, resulting in the formation of hydrophilic protonated merocyanine. This transformation causes the chromophore to switch to a predominantly hydrophilic state, leading to the hydrogel swelling and closing the microchannel due to water absorption. When exposed to blue or white light, the process reverses, causing the gel to contract and the channel to open. While the channel opens within seconds, closing takes several hours, which limits these valves to single-use applications. Ter Schiphorst *et al.*^155^ developed reversible light-responsive hydrogel valves for microfluidic applications, studying the molecular design of spiropyran photoswitches and gel composition. The reversible light-responsive hydrogel valves were integrated within microfluidic channels, allowing reversible and repeatable valve operation in neutral pH under water (see Fig. 2(B)). An innovative combination of graphene oxide (GO) with polymer matrices was investigated in a study by Cheng and colleagues.^156^ They developed self-healing GO-based nanocomposite hydrogels. These hydrogels function as near-infrared (NIR) light-driven valves, capable of undergoing reversible phase transitions and bending when exposed to NIR light (see Fig. 2(C)). An additional advancement in hydrogel actuator was achieved with the development of a remotely controlled hydrogel nanocomposite valves. Satarkar *et al.*^157^ reported a novel approach by dispersing magnetic nanoparticles within temperature-responsive NIPAAm hydrogels to create the nanocomposite hydrogel valve. By applying an alternating magnetic field (AMF), the swelling and collapse of the nanocomposite were remotely controlled. Employing low temperature co-fired ceramic (LTCC) technology, they fabricated a Y-junction ceramic channel where the incorporated ON–OFF nanocomposite valve was controlled by AMF.

Valves and mixing are intricately connected in microfluidic systems, as numerous processes require the merging of multiple channels to combine and homogenize fluids. Within microchannels, laminar flow is the dominant flow regime, and mixing primarily occurs through the process of diffusion. However, in some cases, the mixing is poor due to the low diffusion coefficients of large molecules. To tackle these challenges, micromixers are employed to enhance the mixing efficiency of laminar flows from converging channels. These micromixers are designed to improve the homogenization of fluids, ensuring that the mixing process is more effective and efficient.

Prettyman and colleagues^161^ introduced an innovative micromixer that incorporated stimuli-responsive hydrogel actuators to regulate mixing dynamics based on the chemical properties of fluids. The device, designed in a T-shaped configuration, significantly enhanced mixing efficiency, increasing from 18.3% to 34.5% when transitioning between the contracted and expanded states of the hydrogel. By exploiting the pH-dependent expansion and contraction of hydrogels within microchannels, this mixer provides both active on/off functionality and the inherent advantages of passive mixers. Ter Schiphorst *et al.*^158^ developed a switchable passive mixer designed for controlling mixing and facilitating the easy cleaning of microchannels. By utilizing size-tuneable hydrogels, a passive slanted groove mixer was fabricated that could be switched off by light to alter the mixing behaviour of microfluidics to non-mixed flows. To implement this design for generating a light-responsive micromixer, the spaces between the slanted grooves were filled with a light-responsive hydrogel. This configuration efficiently mixed fluids in the absence of light, as the swollen hydrogel formed the inverse of the scaffold. However, when exposed to visible light, the hydrogel shrank, resulting in an almost smooth channel surface and laminar flow. This work paves the way for multipurpose microfluidic devices, where mixing can be tailored to specific requirements, highlighting the promise of light-induced control in microfluidics (see Fig. 2(D)).

In the field of microfluidic device development, a significant advancement has been made with the invention of a solvent-driven micropump using three-dimensional two-photon microfabrication. Xiong and colleagues^162^ developed an innovative micropump that operates by exploiting the bending behaviour of a hydrogel film in response to asymmetric solvent stimuli. This micropump can absorb and discharge fluid reversibly by alternating the solvent composition between water and ethanol, achieving an impressive response time of 0.17 s. This novel pump addresses the limitations of piezoelectric and electrostatic pumps in implantable or portable drug delivery systems, which require pumping power. In another study, two types of polymeric micropumps were introduced, utilizing temperature-sensitive hydrogels, specifically poly(*N*-isopropylacrylamide) (PNIPAAm), to generate liquid flow. The diffusion micropump featured a single-layer design with a PNIPAAm actuator divided into five segments controlled by resistive heating. Operating in either peristaltic or pulsatile modes, the latter mode provided a higher flow rate and thus enhanced performance.^163^

In addition of the aforementioned applications, hydrogels can encapsulate living cells and biomolecules (*e.g.* proteins and enzymes) offering a supportive environment that replicates natural tissues. This is particularly beneficial in tissue engineering and regenerative medicine, where encapsulated cells/biomolecules can be employed to repair or replace damaged tissues. Thomas Braschler and colleagues^164^ proposed a novel and biocompatible method for the controlled formation of alginate hydrogel by interacting laminar flows of alginate and calcium ions, which enclosed and immobilized yeast cells. By incorporating ethylenediaminetetraacetic acid (EDTA) into the alginate solution and adjusting the concentration of Ca^2+^ ions, they were able to control the hydrogel's growth, allowing precise manipulation of gel expansion and contraction to trap and release cells. Jiao and colleagues^159^ developed a redox-responsive hydrogel integrated into a microfluidic device for reversible protein capture and release. The hydrogel, composed of PNIPAAm with both permanent (*N*,*N*′-methylenebisacrylamide) and redox-responsive (*N*,*N*′-bis(acryloyl) cysteamine) cross-linkers, was fabricated using photopolymerization. The redox-responsive cross-linker enabled the hydrogel to undergo changes in cross-linking density through the cleavage and re-formation of disulfide bonds, resulting in swelling or shrinking behaviour. Rheological measurements confirmed the hydrogel's ability to withstand long-term shear forces in continuous flow conditions. The hydrogel successfully captured and released bovine serum albumin (BSA) labelled with rhodamine B and functionalized with disulfide bonds, achieving an 83.6% release efficiency over three cycles (see Fig. 2(E)). D'eramo *et al.*^82^ fabricated arrays of hydrogel-based cages, up to 7800 cages, that can sequester and release solutes on demand by controlling the temperature, with a density of 44 per mm^2^. The hydrogel walls act as selective membranes, allowing the exchange of small molecules while retaining larger entities like cells. In this study, the key methodological advances included *ex situ* photopatterning with simultaneous cross-linking, grafting, and patterning of the PNIPAAm hydrogel before closing the microfluidic device. *Ex situ* hydrogel synthesis included simultaneous surface-grafting and cross-linking of functionalized polymers *via* thiol–ene click chemistry. The hydrogel was formed on thiol-modified substrates using dithioerythritol as a cross-linker, with polymer solutions in a methanol–butanol mix. This brought the advantages of both chemical cross-linking of the polymer chains and their covalent attachment to the surface, which was accurately controlled. Patterning was achieved through deep UV exposure, by either photolithography or direct laser writing. They demonstrated individual trapping and release of single cells using the caging functionality and integrated the technology into a nucleic acid amplification test for the human synaptojanin 1 gene, suspected to be involved in neurodegenerative diseases. The hydrogel cages successfully confined and amplified the target gene in over 7000 individual compartments. A light-driven micro-heater for single-cell manipulation was developed by Koike's group^160^ using a PNIPAAm hydrogel. They proposed a method that allowed for trapping and mechanically stimulating single motile cells in microchannels, significantly improving throughput and precision compared to traditional methods. The temperature-responsive gel was applied to a glass substrate with light-absorbing micropatterning to achieve localized heating, thereby enhancing response characteristics while minimizing heat dissipation. They also reported the successful integration of multiple actuators on a single chip without unintended activation of neighbouring actuators (see Fig. 2(F)).

Hydrogels are also widely used in drug delivery due to their unique properties. They can be engineered to release drugs at a controlled rate, which helps maintain therapeutic levels of the drug over an extended period. Likewise, they can be designed to respond to specific stimuli present in certain tissues or disease sites. Exploiting these properties, Ha *et al.*^165^ fabricated an electro-responsive hydrogel-based microfluidic actuator platform for precise delivery of plasmonic nanomaterials and targeted photothermal therapy of brain tumours. A conductive hydrogel, made by embedding silver nanowires in collagen I gel, enabled actuation *via* electrical signals. Upon electrical stimulation, the hydrogel converted electrical energy into mechanical energy, releasing nanomaterials. Arg-Gly-Asp (RGD) peptide-conjugated gold nanorods were synthesized for selective destruction of brain tumour cells. The hydrogel, containing endothelial cells and nanomaterials, was delivered to brain tumour cells through a microfluidic setup, followed by laser irradiation for cell destruction. The platform demonstrated controlled delivery and targeted therapy, highlighting advancements in electrically triggered release mechanisms and cancer treatment. In addition to the aforementioned stimuli, smart hydrogels can sense and respond to biological signals, which holds significant promise for drug delivery, medical devices, and diagnostics. Gayet and colleagues^166^ introduced a novel approach using the CRISPR-associated nuclease Cas12a to design nucleic acid-responsive hydrogels, presenting a user-programmable sensor and actuator. The synthesized PEGDA-DNA and polyacrylamide-DNA hydrogels enabled regulated degradation *via* Cas12a. These hydrogels were incorporated into microfluidic paper-based devices that convert DNA inputs into visual or electronic outputs suitable for diagnostic applications. Ambrožič's group^167^ investigated Fe-crosslinked alginate hydrogel for controlled drug release, demonstrated with BSA. The sol–gel transition was triggered by an external electrical signal that changed the oxidation state of Fe ions. Fe(iii) ions formed the hydrogel, while Fe(ii) ions caused its dissolution, controlling the hydrogel thickness by deposition time and current density strength. This process was monitored using a gold electrode inside a microchannel, allowing for continuous or cyclic operation. The designed channel allowed programmable hydrogel dissolution and controlled release of BSA by adjusting electrical conditions.

Following the biological application of hydrogel actuators, Fu *et al.*^168^ developed hydrogels that mimic the colour-changing abilities of natural organisms, such as chameleons. By integrating engineered cardiomyocyte tissues with synthetic inverse opal hydrogel films, hydrogels were synthesized to autonomously regulate their structural colour through cell elongation and contraction. These biohybrid hydrogels can be used in various applications, including dynamic visual displays, intelligent actuators, soft robotic devices, and “heart-on-a-chip” platforms for biological research and drug screening. Sun and colleagues^169^ introduced a novel optical and electrical dual-responsive heart-on-a-chip system, inspired by the vibrant feathers of peacocks. This system used cardiomyocytes hybrid bright MXene structural color hydrogels to evaluate hormone toxicity accurately and efficiently. By incorporating two-dimensional materials like MXene onto photonic crystal array templates, the system mimicked the contraction consistency of actual cardiac tissues, providing a reliable platform for hormone evaluation. Overall, this optical and electrical dual-responsive heart-on-a-chip offers a simple and cost-effective method for rapid screening of various molecules, including hormone drugs, with real-time visualization of cardiomyocyte behaviours. Moreover, Kwon's group^170^ developed a device using a 4-hydroxybutyl acrylate hydrogel to address issues like bubble generation and biocompatibility in electro-responsive smart materials. Operating at low voltages (<1.2 V), the device featured a microfluidic channel with an electroactive hydrogel actuator for particle sorting. It successfully sorted mouse embryoid bodies by size, preserving their pluripotency and ability to differentiate into three germ layers, thereby demonstrating the practical application of smart materials in cell biology.

LoC technologies have made remarkable strides over the past few decades, promising enhanced automation and scalability for analytical and diagnostic applications. Despite these advancements, many innovations have yet to fully realize their potential, especially in automating complex protocols. A significant limitation of current LoC technology is its dependence on MEMS, which require external control mechanisms to operate essential components like pumps and valves. This reliance limits the efficiency of selecting and controlling individual fluid units. A promising solution is logical microfluidics, which aims to use microelectronics for improved on-chip control. Beck *et al.*^171^ developed a chemical volume phase transition transistor (CVPT) employing stimuli-sensitive hydrogels like PNIPAAm to control fluid flow based on specific chemical concentration thresholds. This innovative approach enabled seamless regulation of fluidic circuits using easily manufacturable materials that are already well known within the LoC community. The introduction of CVPT presented a proper solution to several challenges faced by traditional LoC technologies. The CVPT effectively addressed contamination concerns, ensuring signal compliance for cascading operations, and simplifying the complexities associated with chemical signal inversion.

### 2.4 Magnetic material-based actuators

Magnetic materials, a unique class that harness the potential energy stored in magnetic fields to generate mechanical signals, are widely used for precise actuation due to their common response to external magnetic fields, whether from permanent magnets or electromagnets, enabling versatile applications in microfluidics such as pumps, valves, and flow control mechanisms.^103,172^ This capacity enables actuation at multiple levels, from manipulating solid particles and cells, to actuating liquid droplets, and even influencing the flow of the entire fluid stream.^173–175^ The family of magnetic materials encompasses a wide range of substances, including magnetic nanoparticles, ferrofluids, and solid ferromagnetic alloys such as stainless steel, and composite materials.^96,176,177^ One of the main advantages of magnetic separation techniques lies in their simplicity, speed, and cost-effectiveness. Additionally, the ability to rapidly and selectively isolate specific cells or particles is a pivotal step in numerous applications, including biological analyses, food production, chemical processing, and medical diagnostics.^178,179^

**Fig. 3:**
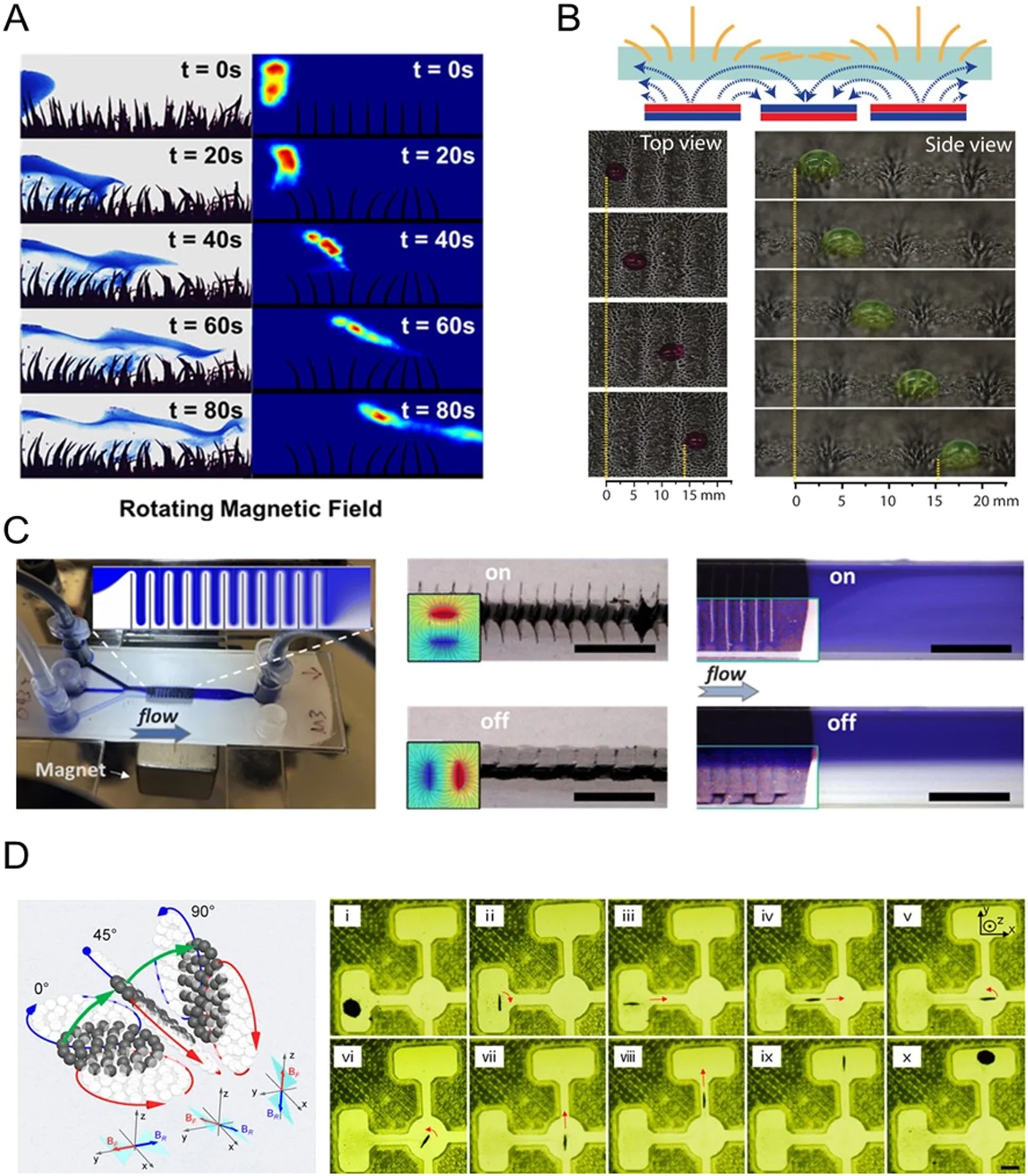
Magnetic material actuators in microfluidic devices. (A) Schematic illustrating experimental and simulation results of particle movement induced by a rotating magnetic field in a unilateral cilia array channel. Reproduced from Sohn *et al.* with permission from Wiley, copyright 2023.^183^ (B) Sketch depicting the orientation of pillars in an NdFeB-powder/soft-silicone liquid-infused soft magnetic carpet exposed to a magnet array (top). Time-lapse of 40 μL droplets moving under a magnetic field on the soft carpet (top view (left) and side views (right)). Reproduced from Demirörs *et al.* with permission from PNAS, copyright 2023.^193^ (C) Experimental setup and images illustrating the “on” and “off” states of magnetic microwalls, composed of magnetic particles embedded in elastomer, for flow control. Reproduced from Broeren *et al.* with permission from RSC, copyright 2023.^191^ (D) Schematic demonstrating the steering of a wheel-like swarm of paramagnetic nanoparticles navigating through a compact cross-shaped narrow channel by adapting to the structure of the channel (i–x). Reproduced from Li *et al.* with permission from Frontiers, copyright 2022.^194^

Magnetic actuation offers advantages for micropump designs, facilitating precise, contactless, and remote-controlled fluid movement. Similarly, magnetic valves benefit from this capability, providing a reliable and non-invasive method for on-demand flow regulation. Several research groups have explored configurations of magnetic-based micropumps and valves. Doganay *et al.*^180^ developed a novel rotating permanent magnetic actuator in 2020, specifically designed to move Fe_3_O_4_–water magnetic nanofluids. This system was used to pump chemicals through micro-sized channels arranged in circular paths. In the same year, Mirkhani *et al.*^181^ investigated a bio-hybrid approach employing magnetostatic bacteria as a self-replicating, biologically based ferrofluid. The bacteria generated a rotating magnetic gradient field to induce directional fluid flow on a PDMS microfluidic device, while a magnetostatic gating field enabled spatially selective actuation. Building on these early developments, Peng *et al.*^182^ introduced a low-cost magnetic micropump in 2021, combining barium ferrite particles with neodymium magnets. This actuation system, integrated into a PMMA device, was driven by the oscillation of magnets in response to audio signals, such as MP3 inputs. More recently, Sohn *et al.*^183^ developed a method for the self-assembly of reprogrammable magnetic cilia arrays. Composed of a mixture of neodymium iron boron (NdFeB) and EcoFlex, a flexible polymer, these cilia were actuated using both striking and rotating magnetic fields, enabling controlled fluid flows within an acrylic channel. This system provided a versatile platform for fluid mixing and pumping, with the added advantage of reprogrammable actuation capabilities (see Fig. 3(A)). Very recently, several new approaches emerged. Wang *et al.*^184^ designed a ferromagnetic ferrofluid as a fluid plug to enable stable flow rates in a PMMA thin film using a dual-rotating magnetic source combined with fixed magnets. Around the same time, Veloso-Fernandez *et al.*^185^ developed a composite material incorporating cobalt ferrite (CoFe_2_O_4_) magnetic nanoparticles within thermo-polyurethane. This composite demonstrated magnetically actuated bending, serving as an efficient valve for on–off switching of fluid flow within a 3D-printed microfluidic channel.

Further expanding the application of magnetic actuation in microfluidics, Zhao *et al.*^186^ introduced reprogrammable magnetic pixel actuators composed of NdFeB nanoparticles embedded in a PDMS matrix. These actuators were utilized to create integrated open-microfluidic functional modules, including on–off valve control. Meanwhile, Li *et al.*^187^ presented a liquid-based magnetic porous membrane incorporating carbonyl iron powder, allowing the generation of highly controllable valves capable of liquid gating, accommodating fluids of varying viscosities.

Similarly, effective mixing is essential for chemical and biochemical interactions, playing a key role in enhancing the performance of advanced LoC devices. However, conventional mixing methods often face challenges in microfluidic environments due to limited turbulence, making the integration of magnetic materials an attractive solution. Recent studies have focused on the innovative use of magnetic particles and composites to enable precise and controllable mixing in microfluidic systems. One approach, demonstrated by Lin *et al.*^188^ involved the use of ferrofluid magnetite particles combined with PEGDA and alginate. This composite material enabled controlled rotation in open microfluidic systems, offering significant potential for biomedical applications. Building on this concept, Sun *et al.*^189^ designed and fabricated a magnetic micromixer that integrated specially designed gold microwires. Their system successfully combined water with ferrofluids in Y-shaped microfluidic channels under uniform magnetic fields. Expanding on these efforts, Feng *et al.*^190^ developed nonspherical magnetic particles containing Fe_3_O_4_ nanoparticles, which allowed precise control over the rotation rate and vortex creation within the fluid. This capability was particularly useful in applications such as the detection of Hg(ii) ions, where effective mixing plays a crucial role in analytical sensitivity. Around the same time, Broeren *et al.*^191^ introduced magneto-actuated microwalls composed of iron powder embedded in a thermoplastic elastomer. These flexible microwalls, integrated into microchannels, enabled on-demand fluid mixing of substances like water and glycerol, further expanding the potential of magnetically driven microfluidic devices (see Fig. 3(C)). Most recently, in 2024, Naghash *et al.*^192^ explored a dynamic mixing strategy using a stainless steel microball within a 3D-printed microfluidic device. The microball was actuated along linear and circular paths using external magnets, ensuring thorough mixing of liquids and demonstrating an innovative, programmable approach to microfluidic mixing. These advancements highlight the rapid evolution of magnetic actuation in microfluidics, demonstrating its versatility in enabling precise, controllable, and efficient fluid manipulation for applications ranging from fluid transport and precision flow control to advanced LoC systems.

In addition to the previously mentioned applications of magnetic materials, on-demand droplet transport is a fundamental capability in microfluidics, supporting a wide range of applications from water collection and environmental monitoring to biomedical detection and chemical analysis. In both digital and open microfluidics, the precise and controlled movement of droplets is essential for tasks such as reagent mixing, sample handling, and droplet-based chemical reactions. The use of magnetic materials has opened promising avenues for enabling externally controlled active droplet transport.^95^ Several research groups have explored innovative configurations for magnetic-based droplet manipulation, demonstrating versatile strategies for controlled movement. Demirörs *et al.*^193^ developed magnetic “carpets” composed of NdFeB particles embedded in EcoFlex, a flexible polymer, enabling precise droplet transport across open microfluidic surfaces (see Fig. 3(B)). Following this concept, Peng *et al.*^195^ introduced a micropillar array made from a composite of liquid silicone and iron powder, providing a multifunctional platform for droplet manipulations, including the microchemical reaction of CaCO_3_ crystallization. Similarly, He *et al.*^196^ studied the movement of droplets using an actuated stainless-steel bead composed of a mixture of carbon, oxygen, chromium, manganese, iron, and nickel, demonstrating effective droplet transport and mixing. In another approach, Kichatov *et al.*^197^ explored the use of iron magnetic nanoparticles dispersed in the continuous phase of an emulsion, enabling the controlled movement of droplets within the flow.

Numerous research groups have explored diverse magnetic-based sorting configurations, developing innovative and efficient methods for the precise separation of biological samples. Yang *et al.*^198^ investigated the use of magnetic nanoparticles in the development of a cell-sorting system, incorporating an array of microcolumns acting as a micro-sieve within a PDMS device to separate target cells from background cells while removing redundant magnetic nanoparticles. Phiphattanaphiphop *et al.*^199^ employed magnetic beads conjugated with antibodies for sperm cell sorting on a glass microfluidic device, targeting the isolation of X-sperm cells from Y-sperm cells. Wang *et al.*^200^ investigated both intra- and extracellular labelling of cells with magnetic nanoparticles to achieve high-performance magnetic cell sorting. This approach focused on the analysis of rare tumour cells and the purification of transfected chimeric antigen receptor (CAR) T cells, a critical step in cell-based immunotherapy. Liu *et al.*^201^ developed a magnetic F-MNP@SiO_2_ coating for droplets, enabling biocompatible sorting of cell-loaded droplets. This method allowed for the encapsulation and sorting of cells within droplet microenvironments, maintaining cell viability and functionality throughout the process. In a similar vein, Nian *et al.*^202^ functionalized streptavidin-coated magnetic beads with an aptamer for the targeted sorting, purification, release, and detection of circulating tumor cells from whole blood samples within a PDMS device. Wang *et al.*^203^ employed the intracellular uptake of biocompatible iron oxide nanoparticles for the analysis of cell proliferation based on magnetization levels within cell cultures. This method facilitated, within a 3D printed device, the sorting of cells based on their phase in the cell division cycle, offering valuable insights into cellular behavior, while also being adaptable to CRISPR-based phenotyping.

Magnetic materials also play a pivotal role in the development of soft- and microrobots, which rely on their ability to perform advanced tasks such as autonomous locomotion, shape morphing, and precise manipulation. These robots use magnetic fields for remote and programmable control, enabling navigation through complex environments and the execution of tasks like targeted drug delivery or microscale assembly.^103^ For instance, Li *et al.*^194^ used superparamagnetic nanoparticles (Fe_12_O_19_Sr) to design fish-like swarm robots capable of multimodal locomotion and load-carrying, highlighting the potential for cooperative robotic systems in biomedical and industrial applications (see Fig. 3(D)). Furthermore, Xu *et al.*^204^ optimized Fe_3_O_4_ nanoparticles to function as microrobots within a 3D-printed microfluidic device, enabling precise manipulation and navigation through microchannels. Similarly, Ku *et al.*^205^ developed a hydrogel hollow fibre doped with carbon nanotubes, Fe_3_O_4_ nanoparticles, and MnO_2_ particles. This composite allowed for the generation of microrobots with dual magnetic and chemical actuation.

The summary of discussed materials, their application, stimuli and fabrication/integration methodologies have been presented in Table 1.

**Table 1:** Functional materials, their application, stimuli and fabrication/integration methods

Material	Application	Stimulus	Fabrication/integration methodology	References
3D printed filter paper	Valve	Manual	Filter paper, polypropylene melted into the paper to create the channel and polylactic acid (PLA) was used to 3D print the valves	Zargaryan *et al.*^122^
Paper	Valve	Gravity	Filter paper, the moisture of the filter paper pushed down the paper cantilever and this made the connection	Kumar *et al.*^123^
Paper	Valve/delay	Mechanical motion and solubility of the sucrose	Dissolvable sucrose delay was integrated into the paper structure; also the expansion of a sponge was used to create a mechanical motion valve	Tu *et al.*^124^
Nitrocellulose, filter paper	ELISA	Manual	Screen-printing polyurethane acrylate (PUA) was used to define the channels in the paper. The paper discs were rotated on each other. Into a sensor disc	Lin *et al.*^121^
PMMA-paper	Valve	Mechanical	CNC micromilling and soft lithography	Hu *et al.*^22^
PDMS prepolymer + Fe_3_O_4_ nanoparticles	Tubing bending + movement of liquids/drops	Magnetic	Magnetic layer inserted into the PDMS	Zhao *et al.*^206^
Pixel magnetic actuator (PDMS, NdFeB NP)	Valves, curation of materials and liquid delivery	Magnetic	Magnetic material immersed into PDMS	Zhao *et al.*^186^
PDMS–ionic polymer–metal composite (IPMC)	Mixing	Physical oscillation-electric signal	PDMS chamber, the IPMC is embedded into the channels	Annabestani *et al.*^40^
Graphene oxide–PDMS	Droplet manipulation	Electric field	Graphene oxide embedded into the PDMS	Basu *et al.*^41^
2-Hydroxyethyl methacrylate (HEMA) + ethylene glycol dimethacrylate (PEGDMA)	Valve	Temperature	Direct photopatterning of liquid phase	Beebe *et al.*^140^
PNIPAAm	Valve	Temperature	Photopolymerization/integration into a polycarbonate	J. Wang *et al.*^70^
PNIPAAm + *N*,*N*-dimethylacrylamide (DMAAm) + *N*,*N*-diethylacrylamide (DEA) + *N*-methylacrylamide (PNMA) + *N*-ethylacrylamide (PNEA)	Valve	Temperature	Radical polymerization/photopatterning	Harmon *et al.*^141^
PNIPAAm	Valve	Temperature	*In situ* photopolymerization	Geiger *et al.*^71^
PNIPAAm	Valve massively integrated into a circuit	Temperature	Photopolymerization/photopatterning	Haefner *et al.*^142^
HEMA	Valve	pH	Liquid phase polymerization	Liu *et al.*^143^
Poly(acrylic acid-2-hydroxyethyl methacrylate) (pAA-HEMA)	Valve, provide the driving pressure to power the dispensing device	pH	*In situ* photopolymerization	Eddington & Beebe,^144^
Phenylboronic acid-based hydrogel (PBA)	Drug delivery	Chemical (glucose)-pH	Free radical polymerization	Baldi *et al.*^145^
PNIPAAm + HEMA	Active wall and delivery piston	Temperature–pH	Photopolymerization	Kim & Beebe^146^
HEMA	Micro check-valve	Mechanical pressure	Tectonic and liquid-phase photopolymerization	Kim & Beebe^147^
PEGDA	Micro check-valve	Mechanical pressure	Optofluidic lithography	Wang *et al.*^148^
PNIPAAm	Valve	Light	Photopolymerization + laser ablation and micro-milling for valve integration	Pandurangan *et al.*^153^
PNIPAAm	Valve	Light	Photopolymerization	Ter Schiphorst *et al.*^155^
Graphene oxide-based nanocomposite of PNIPAAm + poly(*N*,*N* dimethylacrylamide) (PDMAA)	Valve	Light	*In situ* free radical polymerization	Cheng *et al.*^156^
PNIPAAm	On–off valve	Temperature-magnetic	Magnetic particle sonication inside the polymer and photopolymerization	Satarkar *et al.*^157^
PNIPAAm	Microvalve	Light	Localized photo polymerization	Sugiura *et al.*^154^
HEMA	Micromixer	pH	*In situ* photopolymerization	Prettyman & Eddington^161^
PNIPAAm	Micromixer	Light	Photopolymerization	Ter Schiphorst *et al.*^158^
Polyacrylamide (PAAM)	Micropump	Solvent/chemical	Two-photon microfabrication (TPM)	Xiong *et al.*^162^
PNIPAAm	Diffusion-based micropump	Temperature	*In situ* photolithographic polymerisation	Richter *et al.*^163^
Alginate	Cell entrapment and release	Chemical	*In situ* physical polymerization in CaCl_2_	Braschler *et al.*^164^
4-Hydroxybutyl acrylate (4-HBA)	Live cell sorting in cell culture media	Electricity	*In situ* photopolymerization	Kwon *et al.*^170^
Methacrylated gelatin (GelMA),	Soft robotic, heart-on-a-chip platforms	Mechanical	Sacrificial template	Fu *et al.*^168^
PNIPAAm	Cages for cell culture	Temperature	*Ex situ* surface grafting/photopatterning	D'eramo *et al.*^82^
PNIPAAm	Microheater for single-cell manipulation	Light	Photopolimerization/micropatterning	Koike *et al.*^160^
Embedded silver nanowires in collagen I	Delivery of plasmonic nanomaterials/photothermal therapy	Electricity	Silver nanowires were added to collagen I then gelated in 37 °C incubator for 30 min	Ha *et al.*^165^
PEG-DNA + PAM-DNA	Drug release	Biological molecules	Radical-catalysed polymerization	Gayet *et al.*^166^
Alginate	Drug release	Electricity	Electrodeposition	Ambrožič & Plazl^167^
PNIPAAm	Capturing and releasing proteins	Chemical-redox	Photopolymerization	Jiao *et al.*^159^
GelMA	Heart-on-a-chip	Optical–electrical	Photonic crystal (PhC) templates and photopolymerization	Sun *et al.*^169^
PNIPAAm	Planar chemical volume phase transition valve	Chemical	*In situ* polymerization	Beck *et al.*^171^
Ferrofluid NP in PEGDA + alginate	Stirrers in biological applications	Magnetic	Synthesis in microfluidic device: with two phases of oleic acid, Ca^2+^, commercial ferrofluid nanoparticles, and alginate 2%, PEGDA 15%, 1% photoinitiator	Lin *et al.*^188^
Permanent magnetic actuator (PMA) for movement of Fe_3_O_4_–water magnetic nanofluid	Generation of flow	Magnetic	Commercial nanofluid, used in suspension form	Doganay *et al.*^180^
Barium ferrite particles with neodymium magnets	Pump	Magnetic	PMMA chip	Peng *et al.*^182^
Magnetic nanoparticles	Cell-sorting system/micro-sieve	Magnetic	Commercial nanofluid, used in suspension form	Yang *et al.*^198^
NdFeB + EcoFlex magnetic carpets	Droplet movement	Magnetic	Commercial nanofluid, used in suspension form	Demirörs *et al.*^193^
Superparamagnetic nanoparticles (Fe_12_O_19_Sr)	Locomotion and load-carrying capabilities	Magnetic	Commercial superparamagnetic nanoparticles dispersed in water	Li *et al.*^194^
Au microwires	Micromixer	Magnetic	Y-shaped microfluidic device	Sun *et al.*^189^
NdFeB + EcoFlex magnetic cilia	Liquid movement	Magnetic	Acrylic channel NdFeB + EcoFlex	Sohn *et al.*^183^
Magnetic nonspherical particles containing Fe_3_O_4_ nanoparticles	Mixer	Magnetic	Microfluidics-based formation of droplets	Feng *et al.*^190^
Magnetic (FMNP@SiO_2_) cover of droplets	Sorting of cell-loaded droplets	Magnetic	Microfluidics-based formation of droplets	Liu *et al.*^201^
Stainless steel bead (C, O, Cr, Mn, Fe, Ni)	Movement and mixing of drops	Magnetic	Open fluidics	He *et al.*^196^
Magnetic SIBS (iron powder) microwalls	Mixer	Magnetic	Hot embossing with magnetic SIBS pre-mixed with magnetic particles bound to the microfluidic device	Broeren *et al.*^191^
Micropillar array (liquid silicone, silicone oil, and iron powder)	Multi-functional droplet manipulation	Magnetic	Open fluidics	Peng *et al.*^195^
Iron magnetic nanoparticles	Controlled movement of droplets in flow	Magnetic		Kichatove *et al.*^197^
Ferrofluid	Valve and fluid movement	Magnetic		Wang *et al.*^184^
EGaIn@Fe, liquid metal	2D and 3D movement in channels	Magnetic		Ku *et al.*^205^
Magnetotactic bacteria and iron oxide nanoparticles	Rotating gradient field that generates directional flow; Magnetostatic gating field that enables spatially selective actuation	Magnetic	Commercial iron NP, bacteria were cultured	Mirkhani *et al.*^181^
Magnetic beads conjugated with antibodies	Sorting and separation of sperm cells	Magnetic	Commercial magnetics particle beads	Phiphattanaphiphop *et al.*^199^
Magnetic nanoparticle labelled cells	Cell sorting for analysis of rare tumour cells and purification of transfected CAR T cells	Magnetic	Commercial antibody-conjugated magnetic nanoparticles	Wang *et al.*^200^
Streptavidin coated magnetic beads functionalized with aptamer	Sorting, purification, release, and detection of circulating tumour cells from blood	Magnetic	Commercial streptavidin magnetic beads	Nian *et al.*^202^
Fe_3_O_4_ nanoparticles for microrobots	Robotic	Magnetic		Y. Xu *et al.*^204^
TPU/CoFe_2_O_4_ composites	Valves, resistance of flow	Magnetic	CoFe_2_O_4_ nanoparticles added to thermo-polyurethane polymer synthesis	Veloso-Fernández *et al.*^185^
Magnetic stainless steel microball	Micromixer	Magnetic		Hajihadi Naghash *et al.*^192^
Intracellular uptake of iron oxide nanoparticles with biocompatible shell	Analysis of cell proliferation based on magnetization levels in the cell culture, sorting between phases of cell division, adaptable to *in vivo* CRISPR phenotyping	Magnetic	Commercial carboxylated MNPs and Cy5-conjugated MNPs	Wang *et al.*^203^

## 3. Next generation actuators

The field of functional materials is constantly expanding, offering an ever-growing range of possibilities for their application in microfluidics. In this review, we have highlighted several classes of materials currently used as actuators in microfluidic devices, demonstrating their transformative potential in various applications. Beyond these established options, ongoing research into both conventional and novel materials is uncovering new opportunities for their use as microactuators. This rapidly evolving field of study is bound to have a significant impact on microfluidics in the near future, driving innovation in device functionality, responsiveness, and integration.

A particularly promising class of functional materials is liquid crystals (LCs), which have attracted increasing attention due to their anisotropic viscoelastic properties and long-range orientational order. As soft yet highly responsive materials, LCs can dynamically adjust their physical and chemical properties in response to external stimuli, making them excellent candidates for actuation in microfluidic devices.^207,208^ When incorporated into polymer networks, these materials form liquid crystal polymers (LCPs) which can be further classified as liquid crystal elastomers (LCEs) or liquid crystal networks (LCNs) upon crosslinking. These crosslinked LCPs are particularly suitable for actuator applications, as they undergo substantial and reversible shape changes as they transit from the ordered liquid crystal phase to the disordered isotropic phase.^208^ Their adaptability extends to a range of soft robotic applications, including microgrippers, biomimetic devices, and portable haptic systems.^209,210^ Additionally, cholesteric liquid crystals, which have nano-helical structures similar to those found in biological organisms, can be integrated into actuators to generate vivid structural colour changes in response to environmental stimuli.^211^ Modulated at the nanoscale, these structural colours, can selectively reflect specific wavelengths of light, providing unique visual feedback mechanisms that go beyond the limits of conventional colour recognition.^212^ Recent advances have demonstrated that LCN actuators can be driven by various external stimuli, such as heat, light, humidity, and electrical signals, further expanding their applicability in microfluidic and soft robotic systems.^213–216^ The ability of LCEs and LCNs to be processed into various morphologies, including thin films, fibres, monolithic structures, and composite materials, enhances their potential for seamless integration into microfluidic platforms.

**Fig. 4:**
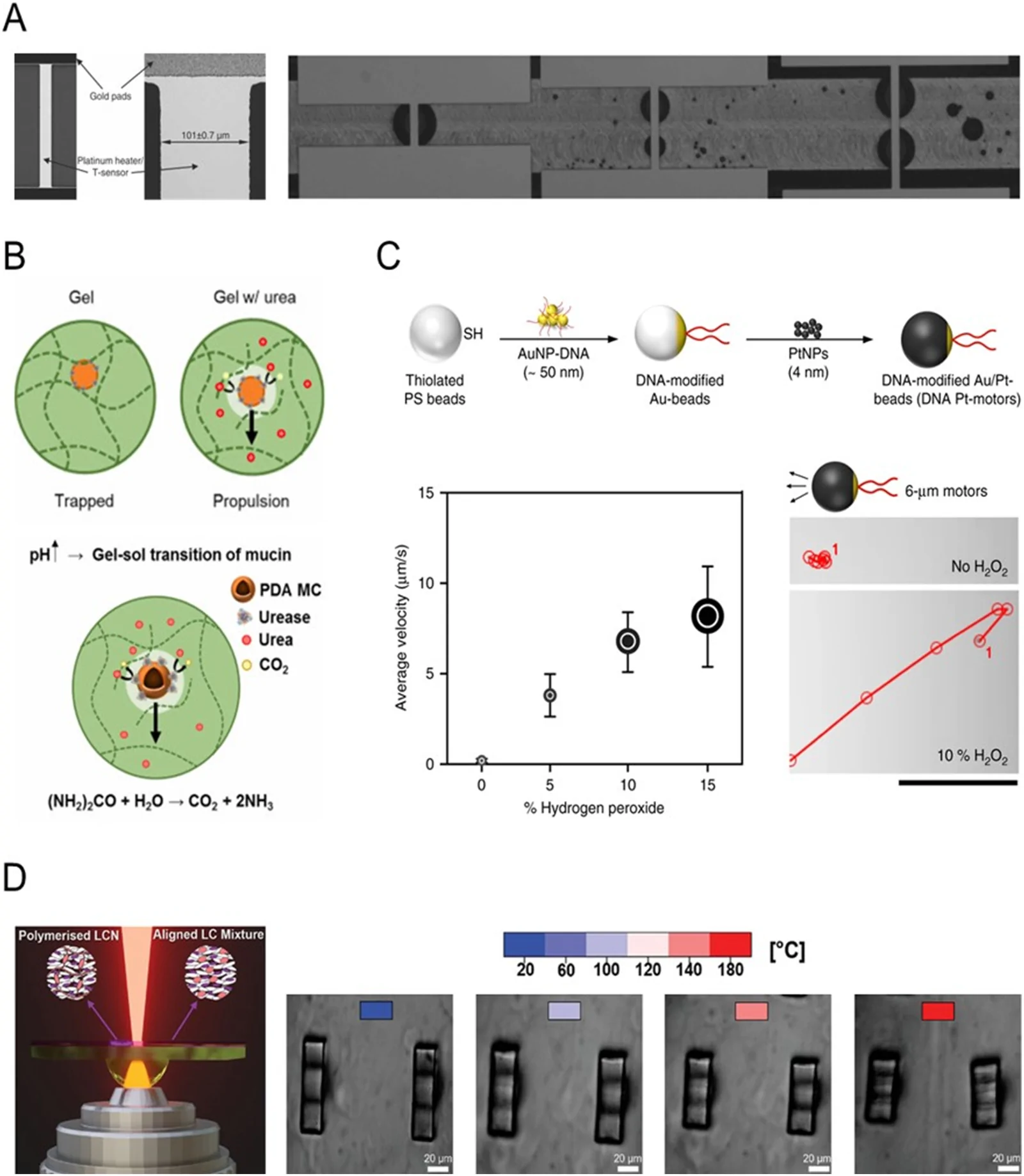
Novel functional materials with potential use as actuators in microfluidic devices. (A) Optical micrograph of a fabricated platinum heater (left) and photographs of vapor bubbles in hot deionized (DI) water with 0.5, 1.0- and 1.5 mm heaters at applied heating powers of 198, 332 and 446 mW, respectively. Reproduced from Schepperle *et al.* with permission from Elsevier, copyright 2022.^234^ (B) Schematic illustrating the mechanism of a urease-based micromotor for controlled local pH increase. Reproduced from Choi *et al.* with permission from Elsevier, copyright 2022.^239^ (C) Schematic of the synthesis and velocity analysis of DNA-motors powered with hydrogen peroxide. Reproduced from Draz *et al.* with permission from Nature, copyright 2018.^240^ (D) Two-photon polymerization of microstructures composed of liquid crystal networks exhibiting thermos-responsive contraction. Reproduced from Donato *et al.* with permission from Wiley, copyright 2023.^245^

Nanoparticles have also emerged as a versatile class of functional materials with significant potential in microfluidic applications. While magnetic nanoparticles have already been explored for actuation and manipulation purposes, polymeric and bio-inspired nanoparticles offer additional functionalities, including biosensing and controlled cargo delivery and release mechanisms.^217–221^ These nanoparticles can encapsulate various payloads, such as drugs or biomolecules, and respond to specific stimuli for targeted release, making them valuable for biomedical microfluidic applications.^222–224^ In addition, metallic nanoparticles, particularly gold, silver and platinum, have been extensively studied due to their unique plasmonic properties, which enable them to function as highly sensitive elements in optical sensing platforms.^225,226^ Their strong interaction with light enables precise detection mechanisms but also facilitates their role as efficient microheaters when exposed to electromagnetic radiation.^227–229^ The heating capabilities of metallic nanoparticles can be exploited for localized temperature modulation within microfluidic environments to drive fluid displacement, enhance mixing, control the generation of bubbles or enable localized thermal processing, resulting in exciting applications such as molecular biology or the development of electromechanical devices (see Fig. 4(A)).^230–234^

Taking direct inspiration from biochemical processes found inside cells, another rapidly advancing category of functional materials is bio-derived micromotors and robots. For instance, enzyme-based micromotors use biochemical reactions, such as urease and glucose oxidase reactions to catalyse reactions and generate motion. The controlled conversion of chemical energy into mechanical movement provides these micromotors with exceptional adaptability and efficiency (see Fig. 4(B)).^235–239^ More recently, DNA oligonucleotides have been explored for the generation of DNA-based motors, using programmable hybridization mechanisms to achieve precise movement control (see Fig. 4(C)).^240–242^ Additionally, whole microorganisms have been investigated as bio-actuators, offering unique advantages in terms of self-propulsion and responsiveness to environmental stimuli. In particular, T4 bacteriophages have been used to label and select magnetic micromotors, while light-induced bacterial movement has been exploited to generate micro-reactors in fluidic environments.^243,244^ These biological micromotors and robots offer an innovative approach to microfluidic actuation, combining the precision of synthetic control with the dynamic adaptability of living systems.

In addition to the search for novel materials, considerable investigations have focused on the methods for constructing, polymerizing, and integrating these materials into functional systems. Micro- and nanoscale structuring has proven to be critical in achieving functionalities that are not achievable at the macroscale. Direct laser writing *via* two-photon polymerization (DLW-2PP) is an advanced 3D nanofabrication technique that uses a focused femtosecond laser to initiate polymerization only at the focal point of the laser, where two photons are absorbed in quick succession, *via* a short-lived virtual state. This nonlinear process enables precise structuring inside photosensitive materials, allowing for the creation of complex 3D micro- and nanostructures. The technique achieves voxel sizes as small as 100 nanometers in width, offering exceptional resolution beyond the diffraction limit. 2PP-DLW is widely used in fields like photonics, biomedicine, and microfluidics for fabricating custom-designed microdevices.^246–248^ This method has emerged as a highly effective technique for inducing locally selective polymerization in various stimuli-responsive materials.^249^ This method offers exceptional resolution and an unparalleled degree of freedom in structural design, enabling the fabrication of microactuators capable of performing advanced tasks such as walking, grasping, swimming, and drug delivery. DLW has become the method of choice for 3D printing precise and complex geometries in micro- and nanoscale dimensions, enabling the production of both 3D and 4D microstructures.^250–252^ Shape memory polymers (SMPs),^253^ a class of smart materials capable of controlled shape changes upon external stimuli such as light, temperature,^254–256^ or chemical inputs such as pH or sugar,^251,257^ have garnered particular interest in this context.^255^ Microscale 4D printing using DLW has already been demonstrated for hydrogels, elastomeric liquid crystals, and composite materials, paving the way for multifunctional microactuators. The ability to induce nanostructuring in these materials enables them to respond to multiple stimuli through a single input, directly linked to the nanoscale geometries achieved during fabrication. For instance, this structuring can be used to create microstructures with photonic capabilities enabling self-reporting of material actuation, providing real-time feedback by changing optical properties in response to external stimuli such as temperature or vapor, further enhancing their functionality in smart sensing and adaptive systems (see Fig. 4(D)).^245,258–261^ Simultaneously, it allows for the generation of microactuators composed of both conventional and novel materials, such as hydrogels and ionogels,^256,262–264^ with the potential for seamless integration into future microfluidic devices. By combining innovative materials and advanced fabrication techniques, these developments promise to further increase the versatility and functionality of microfluidic technologies.

## 4. Conclusions

The integration of actuators into microfluidic systems has significantly advanced the miniaturization of fluidic technologies, enabling the execution of complex tasks on an exceptionally small scale. Actuators, which convert various forms of energy into mechanical motion, are essential for controlling fluid flow within microfluidic devices. This precise control is crucial for achieving accurate measurements and facilitating complex analyses. This review has examined recent advancements in actuation technologies, with particular emphasis on the materials employed in these systems. A range of functional materials has been identified, including paper, PDMS, hydrogels, magnetic materials, and their hybrids, all of which are currently utilized in microfluidic devices. The suitability of each material for various applications has been evaluated, highlighting their versatility across multiple contexts. Additionally, methods for fabricating and integrating these materials into microfluidic systems, as well as the stimuli that activate them, have been discussed. This review highlights their role in key microfluidic components, such as micropumps, micromixers, microvalves, and other essential components for precisely controlling liquids though the microchannels. Moreover, several classes of materials with transformative potential have been identified, including bio-derived micromotors and robots, metallic, polymeric, and bio-inspired nanoparticles, as well as liquid crystals. These materials show considerable promise as actuators within microfluidic channels, offering new avenues for innovation.

In addition to exploring novel materials, significant efforts have been dedicated to developing methods for fabricating, polymerizing, and integrating these materials into functional systems. A notable technique is direct laser writing, particularly through two-photon polymerization, which has proven highly effective for locally selective polymerization in stimuli-responsive materials. This method enables the creation of microactuators capable of performing advanced tasks such as walking, grasping, swimming, and drug delivery. By combining innovative materials with advanced fabrication techniques, these developments are set to enhance the versatility and functionality of microfluidic technologies, paving the way for exciting new applications and future advancements in the field.

## Author contributions

SI: literature search, conceptualization, writing and correcting of original manuscript. IPR: literature search, writing of original manuscript. EAH: literature search, writing of original manuscript. DP: literature search, writing of original manuscript. LF: literature search, correction of original manuscript. CD: literature search, correction of original manuscript. LBD: conceiving the original idea of the review, conceptualization, correction of original manuscript. FBL: conceiving the original idea of the review, conceptualization, supervision, correction of original manuscript.

## Conflicts of interest

There are no conflicts of interest.

## Data Availability

No primary research results, software or code have been included and no new data were generated or analysed as part of this review.

## References

[cit1] Erickson D., Li D. (2004). Anal. Chim. Acta.

[cit2] Hilber W. (2024). Appl. Phys. A: Mater. Sci. Process..

[cit3] Narayanamurthy V., Jeroish Z. E., Bhuvaneshwari K. S., Bayat P., Premkumar R., Samsuri F., Yusoff M. M. (2020). RSC Adv..

[cit4] Hsu Y. C., Hsu J. L., Le N. B. (2009). Microsyst. Technol..

[cit5] Hwang Y., Candler R. N. (2017). Lab Chip.

[cit6] Wang T., Chen J., Zhou T., Song L. (2018). Micromachines.

[cit7] Lin Y., Gerfen G. J., Rousseau D. L., Yeh S. R. (2003). Anal. Chem..

[cit8] Clegg D. L. (1950). Anal. Chem..

[cit9] Mansur E. A., Mingxing Y., Yundong W., Youyuan D. (2008). Chin. J. Chem. Eng..

[cit10] Li Z., Zhang B., Dang D., Yang X., Yang W., Liang W. (2022). Sens. Actuators, A.

[cit11] Antognoli M., Tomasi Masoni S., Mariotti A., Mauri R., Salvetti M. V., Brunazzi E., Galletti C. (2022). Micromachines.

[cit12] Wang L., Liu D., Wang X., Han X. (2012). Chem. Eng. Sci..

[cit13] Hessel V., Löwe H., Schönfeld F. (2005). Chem. Eng. Sci..

[cit14] Ward K., Fan Z. H. (2015). J. Micromech. Microeng..

[cit15] Waheed A. , Buyong M. R. and Wee M. M. R., in Proceedings - 2023 IEEE Regional Symposium on Micro and Nanoelectronics, RSM 2023, Institute of Electrical and Electronics Engineers Inc., 2023, pp. 142–144

[cit16] Channon R. B., Menger R. F., Wang W., Carrão D. B., Vallabhuneni S., Kota A. K., Henry C. S. (2021). Microfluid. Nanofluid..

[cit17] Komiyama R. , Miyashita H., Kageyama T., Ohmi K., Lee S.-S. and Okura H., A Microfluidic Device Fully Integrated with Three pH Sensing Electrodes and Passive Mixer for Nanoparticle Synthesis, IEEE, 2015

[cit18] Li W., Chu W., Yin D., Liang Y., Wang P., Qi J., Wang Z., Lin J., Wang M., Wang Z., Cheng Y. (2020). Appl. Phys. A: Mater. Sci. Process..

[cit19] Qi J., Li W., Chu W., Yu J., Wu M., Liang Y., Yin D., Wang P., Wang Z., Wang M., Cheng Y. (2020). Micromachines.

[cit20] Shakeri A., Jarad N. A., Leung A., Soleymani L., Didar T. F. (2019). Adv. Mater. Interfaces.

[cit21] Hassan S. U., Zhang X. (2020). Biosensors.

[cit22] Hu W. P., Lai Y. F., Vu C. A., Tsao C. W., Pan S. C., Cheng C. M., Chen W. Y. (2023). Talanta.

[cit23] Yetisen A. K., Akram M. S., Lowe C. R. (2013). Lab Chip.

[cit24] Abate M. F., Ahmed M. G., Li X., Yang C., Zhu Z. (2020). Lab Chip.

[cit25] Patko D., Gunatilake U. B., Gonzalez-Gaya B., Dupuy L. X., Basabe-Desmonts L., Benito-Lopez F. (2024). Soil Biol. Biochem..

[cit26] Goral V. N., Tran E., Yuena P. K. (2015). Biomicrofluidics.

[cit27] Yu Y., Yue T., Liu N., Liu Y., Gao S., Gu S., Zhou Y., Peng Y. (2021). Sens. Actuators, B.

[cit28] Aghajanloo B., Losereewanich W., Pastras C. J., Inglis D. W. (2024). Biomicrofluidics.

[cit29] Jang I., Carraõ D. B., Menger R. F., Moraes De Oliveira A. R., Henry C. S. (2020). ACS Sens..

[cit30] Laurenciano C. J. D., Tseng C. C., Chen S. J., Lu S. Y., Tayo L. L., Fu L. M. (2021). Talanta.

[cit31] Anderson J. R., Chiu D. T., Jackman R. J., Chemiavskaya O., McDonald J. C., Wu H., Whitesides S. H., Whitesides G. M. (2000). Anal. Chem..

[cit32] Fujii T. (2002). Microelectron. Eng..

[cit33] Zuo P., Li X., Dominguez D. C., Ye B. C. (2013). Lab Chip.

[cit34] Mitra S. K. and Chakraborty S., Microfluidics and Nanofluidics Handbook: Fabrication, Implementation, and Applications, CRC Press/Taylor and Francis, 2012

[cit35] You J. B., Kang K., Tran T. T., Park H., Hwang W. R., Kim J. M., Im S. G. (2015). Lab Chip.

[cit36] Lee B., Kim M., Oh S., Bi Lee D., Lee S. G., Min Kim H., Kim K. H., Song J., Lee C. S. (2023). Chem. Eng. Sci..

[cit37] Grossmann G., Guo W. J., Ehrhardt D. W., Frommer W. B., Sit R. V., Quake S. R., Meier M. (2011). Plant Cell.

[cit38] Dou M., Dominguez D. C., Li X., Sanchez J., Scott G. (2014). Anal. Chem..

[cit39] Zhu J., Wang Y., Tang S., Su H., Wang X., Du W., Wang Y., Liu B. F. (2023). Micromachines.

[cit40] Annabestani M., Azizmohseni S., Esmaeili-Dokht P., Bagheri N., Aghassizadeh A., Fardmanesh M. (2020). J. Microelectromech. Syst..

[cit41] Basu M., Parihar V., Lincon A., Joshi V. P., Das S., DasGupta S. (2021). Chem. Eng. Sci..

[cit42] Subandi A., Buyong M. R., Hamzah A. A., Majlis B. Y., Raub A. A. M., Pawinanto R. E., Mulyanti B., Yunas J. (2024). Sens. Actuators, A.

[cit43] Angelini A., Agero U., Ferrarese Lupi F., Fretto M., Pirri F., Frascella F. (2020). Soft Matter.

[cit44] Xu L., Wang A., Li X., Oh K. W. (2020). Biomicrofluidics.

[cit45] Wang P., Yuan S., Yang N., Oppong P. K. (2021). J. Micromech. Microeng..

[cit46] Li G., Luo Y., Chen Q., Liao L., Zhao J. (2012). Biomicrofluidics.

[cit47] Alvarez-Braña Y., Etxebarria-Elezgarai J., Ruiz de Larrinaga-Vicente L., Benito-Lopez F., Basabe-Desmonts L. (2021). Sens. Actuators, B.

[cit48] Hosokawa K. (2021). Anal. Sci..

[cit49] Shen Y., Tanaka N., Yamazoe H., Furutani S., Nagai H., Kawai T., Tanaka Y. (2020). PLoS One.

[cit50] Ahmed E. M. (2015). J. Adv. Res..

[cit51] Ogawa I., Yamano H., Miyagawa K. (1993). J. Appl. Polym. Sci..

[cit52] Bashir S., Hina M., Iqbal J., Rajpar A. H., Mujtaba M. A., Alghamdi N. A., Wageh S., Ramesh K., Ramesh S. (2020). Polymers.

[cit53] Ho T. C., Chang C. C., Chan H. P., Chung T. W., Shu C. W., Chuang K. P., Duh T. H., Yang M. H., Tyan Y. C. (2022). Molecules.

[cit54] Khan M. U. A., Aslam M. A., Bin Abdullah M. F., Al-Arjan W. S., Stojanovic G. M., Hasan A. (2024). Arabian J. Chem..

[cit55] Gao Y., Song J., Li S., Elowsky C., Zhou Y., Ducharme S., Chen Y. M., Zhou Q., Tan L. (2016). Nat. Commun..

[cit56] Gunatilake U. B., Caballe-Abalos A., Garcia-Rey S., Mercader-Ruiz J., Basabe-Desmonts L., Benito-Lopez F. (2023). Adv. Mater. Interfaces.

[cit57] Merchant S. A., Iran T. O., Meredith M. T., Cline T. C., Glatzhofer D. T., Schmidtke D. W. (2009). Langmuir.

[cit58] Garcia-Rey S. , Ojeda E., Gunatilake U. B., Basabe-Desmonts L. and Benito-Lopez F., MicroTAS 2021 - 25th International Conference on Miniaturized Systems for Chemistry and Life Sciences, 2021, pp. 1547–1548

[cit59] Verbeke C. S., Gordo S., Schubert D. A., Lewin S. A., Desai R. M., Dobbins J., Wucherpfennig K. W., Mooney D. J. (2017). Adv. Healthcare Mater..

[cit60] He M., Nandu N., Uyar T. B., Royzen M., Yigit M. V. (2020). Chem. Commun..

[cit61] Kurt E., Segura T. (2022). Adv. Healthcare Mater..

[cit62] Ye Y., Xu P., Li C., Jin S., Hu J., Fang Y., Zhu K., Xu G., Han Z., Zhang Z., Wu N., Jiang P., Bao Z., Zhou P., Zhang C. (2023). Chem. Eng. J..

[cit63] Nicodemus G. D., Bryant S. J. (2008). Tissue Eng., Part B.

[cit64] Burnett K., Edsinger E., Albrecht D. R. (2018). Commun. Biol..

[cit65] Farasati Far B., Safaei M., Nahavandi R., Gholami A., Naimi-Jamal M. R., Tamang S., Ahn J. E., Ramezani Farani M., Huh Y. S. (2024). ACS Omega.

[cit66] Hu X., Lei B., Li S., Chen L., Li Q. (2023). Responsive Mater..

[cit67] Ching S. H., Bansal N., Bhandari B. (2017). Crit. Rev. Food Sci. Nutr..

[cit68] Lei Z., Wang Q., Sun S., Zhu W., Wu P. (2017). Adv. Mater..

[cit69] Liu X., Gao M., Chen J., Guo S., Zhu W., Bai L., Zhai W., Du H., Wu H., Yan C., Shi Y., Gu J., Qi H. J., Zhou K. (2022). Adv. Funct. Mater..

[cit70] Wang J., Chen Z., Mauk M., Hong K. S., Li M., Yang S., Bau H. H. (2005). Biomed. Microdevices.

[cit71] Geiger E. J., Pisano A. P., Svec F. (2010). J. Microelectromech. Syst..

[cit72] Carayon I., Gaubert A., Mousli Y., Philippe B. (2020). Biomater. Sci..

[cit73] Sershen S. R., Mensing G. A., Ng M., Halas N. J., Beebe D. J., West J. L. (2005). Adv. Mater..

[cit74] Kato E. (2000). J. Chem. Phys..

[cit75] Gupta P., Vermani K., Garg S. (2002). Drug Discovery Today.

[cit76] Wei W., Li J., Qi X., Zhong Y., Zuo G., Pan X., Su T., Zhang J., Dong W. (2017). Carbohydr. Polym..

[cit77] Miyata T., Uragami T., Nakamae K. (2002). Adv. Drug Delivery Rev..

[cit78] Le X., Lu W., Zhang J., Chen T. (2019). Adv. Sci..

[cit79] Beck A., Obst F., Gruner D., Voigt A., Mehner P. J., Gruenzner S., Koerbitz R., Shahadha M. H., Kutscher A., Paschew G., Marschner U., Richter A. (2023). Adv. Mater. Technol..

[cit80] Li A. , Lee J., Gray B. L. and Li P. C. H., Microfluidics, BioMEMS, and Medical Microsystems X, 2012, vol. 8251, p. 82510Z

[cit81] Yuk H., Lin S., Ma C., Takaffoli M., Fang N. X., Zhao X. (2017). Nat. Commun..

[cit82] D'eramo L., Chollet B., Leman M., Martwong E., Li M., Geisler H., Dupire J., Kerdraon M., Vergne C., Monti F., Tran Y., Tabeling P. (2018). Microsyst. Nanoeng..

[cit83] Kwon G. H., Jeong G. S., Park J. Y., Moon J. H., Lee S. H. (2011). Lab Chip.

[cit84] Herrlich S., Spieth S., Messner S., Zengerle R. (2012). Adv. Drug Delivery Rev..

[cit85] Mah E., Ghosh R. (2013). Processes.

[cit86] Koetting M. C., Peters J. T., Steichen S. D., Peppas N. A. (2015). Mater. Sci. Eng., R.

[cit87] Yang L., Liu Y., Shou X., Ni D., Kong T., Zhao Y. (2020). Nanoscale.

[cit88] Tolabi H., Davari N., Khajehmohammadi M., Malektaj H., Nazemi K., Vahedi S., Ghalandari B., Reis R. L., Ghorbani F., Oliveira J. M. (2023). Adv. Mater..

[cit89] Frent O. D., Vicas L. G., Duteanu N., Morgovan C. M., Jurca T., Pallag A., Muresan M. E., Filip S. M., Lucaciu R. L., Marian E. (2022). Int. J. Mol. Sci..

[cit90] Yao C., Liu Z., Yang C., Wang W., Ju X. J., Xie R., Chu L. Y. (2015). Adv. Funct. Mater..

[cit91] Breuer L., Raue M., Strobel M., Mang T., Schöning M. J., Thoelen R., Wagner T. (2016). Phys. Status Solidi A.

[cit92] Pamme N. (2006). Lab Chip.

[cit93] Kim S.-E., Van Tieu M., Hwang S. Y., Lee M.-H. (2020). Micromachines.

[cit94] Yang R.-J., Hou H.-H., Wang Y.-N., Fu L.-M. (2016). Sens. Actuators, B.

[cit95] Al-Hetlani E., Amin M. O. (2019). Microchim. Acta.

[cit96] Suwa M., Tsukahara S., Watarai H. (2023). Lab Chip.

[cit97] Nguyen N.-T. (2012). Microfluid. Nanofluid..

[cit98] Chen X., Zhang L. (2017). Microchim. Acta.

[cit99] Sahadevan V., Panigrahi B., Chen C.-Y. (2022). Micromachines.

[cit100] Paryab A., Saghatchi M., Zarin B., Behsam S., Abdollahi S., Malek Khachatourian A., Toprak M. S., Amukarimi S., Qureshi A., Niazi J. H. (2024). Rev. Chem. Eng..

[cit101] Zhang Y., Nguyen N.-T. (2017). Lab Chip.

[cit102] Bacchetti A., Lloyd P., Taccola S., Fakhoury E., Cochran S., Harris R. A., Valdastri P., Chandler J. H. (2022). Front. Robot. AI.

[cit103] Gray B. L. (2014). J. Electrochem. Soc..

[cit104] Zhang C., Xing D., Li Y. (2007). Biotechnol. Adv..

[cit105] Niedl R. R., Beta C. (2015). Lab Chip.

[cit106] Sarabi M. R., Yigci D., Alseed M. M., Mathyk B. A., Ata B., Halicigil C., Tasoglu S. (2022). iScience.

[cit107] Yamada K., Henares T. G., Suzuki K., Citterio D. (2015). Angew. Chem..

[cit108] Singh A. T., Lantigua D., Meka A., Taing S., Pandher M., Camci-Unal G. (2018). Sensors.

[cit109] Böhm A., Trosien S., Avrutina O., Kolmar H., Biesalski M. (2018). Front. Chem..

[cit110] Xie L., Zi X., Zeng H., Sun J., Xu L., Chen S. (2019). Anal. Chim. Acta.

[cit111] Carrilho E., Martinez A. W., Whitesides G. M. (2009). Anal. Chem..

[cit112] Catalan-Carrio R., Akyazi T., Basabe-Desmonts L., Benito-Lopez F. (2021). Sensors.

[cit113] Kumar S., Kaushal J. B., Lee H. P. (2024). Biosensors.

[cit114] Ongaro A. E., Ndlovu Z., Sollier E., Otieno C., Ondoa P., Street A., Kersaudy-Kerhoas M. (2022). Lab Chip.

[cit115] Bhattacharya S. , Kumar S. and Agarwal A. K., Advanced Functional Materials and Sensors Paper Microfluidics, Springer, 2019

[cit116] Agustini D., Caetano F. R., Quero R. F., Fracassi Da Silva J. A., Bergamini M. F., Marcolino-Junior L. H., De Jesus D. P. (2021). Anal. Methods.

[cit117] Agustini D., Bergamini M. F., Marcolino-Junior L. H. (2016). Lab Chip.

[cit118] Nilghaz A., Wicaksono D. H. B., Gustiono D., Abdul Majid F. A., Supriyanto E., Abdul Kadir M. R. (2012). Lab Chip.

[cit119] Chang C. H., Wang C. L., Li B. R. (2023). Biosens. Bioelectron..

[cit120] AlMashrea B. A., Almehdi A. M., Damiati S. (2024). Front. Bioeng. Biotechnol..

[cit121] Lin D., Li B., Fu L., Qi J., Xia C., Zhang Y., Chen J., Choo J., Chen L. (2022). Microsyst. Nanoeng..

[cit122] Zargaryan A., Farhoudi N., Haworth G., Ashby J. F., Au S. H. (2020). Sci. Rep..

[cit123] Kumar A., Heidari-Bafroui H., Charbaji A., Rahmani N., Anagnostopoulos C., Faghri M. (2021). Chem. Proc..

[cit124] Tu D., Holderby A., Dean J., Mabbott S., Coté G. L. (2021). Anal. Chem..

[cit125] Atabakhsh S., Haji Abbasali H., Jafarabadi Ashtiani S. (2024). Talanta.

[cit126] Braunger M. L., Fier I., Rodrigues V., Arratia P. E., Riul A. (2020). Chemosensors.

[cit127] Nakatani M., Tanaka Y., Okayama S., Hashimoto M. (2020). Electrophoresis.

[cit128] Wang A. , Boroujeni S. M., Andreadis S. T. and Oh K. W., MicroTAS 2020 - 24th International Conference on Miniaturized Systems for Chemistry and Life Sciences, 2020, pp. 713–714

[cit129] Le T. N., Nguyen V. A., Bach G. L., Tran L. D., Cao H. H. (2020). Adv. Polym. Technol..

[cit130] Qaiser N., Khan S. M., Babatain W., Nour M., Joharji L., Shaikh S. F., Elatab N., Hussain M. M. (2023). J. Micromech. Microeng..

[cit131] Venzac B., Liu Y., Ferrante I., Vargas P., Yamada A., Courson R., Verhulsel M., Malaquin L., Viovy J. L., Descroix S. (2020). Microsyst. Nanoeng..

[cit132] Sesen M., Rowlands C. J. (2021). Microsyst. Nanoeng..

[cit133] Kalulu M., Chilikwazi B., Hu J., Fu G. (2024). Macromol. Rapid Commun..

[cit134] Ter Schiphorst J., Saez J., Diamond D., Benito-Lopez F., Schenning A. P. H. J. (2018). Lab Chip.

[cit135] Song Y., Zhou Y., Zhang K., Fan Z., Zhang F., Wei M. (2024). Lab Chip.

[cit136] Chen W., Wen Y., Fan X., Sun M., Tian C., Yang M., Xie H. (2021). J. Mater. Chem. B.

[cit137] Zhao Z., Li G., Ruan H., Chen K., Cai Z., Lu G., Li R., Deng L., Cai M., Cui W. (2021). ACS Nano.

[cit138] Paggi C. A., Hendriks J., Karperien M., Le Gac S. (2022). Lab Chip.

[cit139] Cassel de Camps C., Mok S., Ashby E., Li C., Lépine P., Durcan T. M., Moraes C. (2023). Lab Chip.

[cit140] Beebe D. J., Moore J. S., Bauer J. M., Yu Q., Liu R. H., Devadoss C., Jo B.-H. (2000). Nature.

[cit141] Harmon M. E., Tang M., Frank C. W. (2003). Polymer.

[cit142] Haefner S., Koerbitz R., Frank P., Elstner M., Richter A. (2018). Adv. Mater. Technol..

[cit143] Liu R. H., Yu Q., Beebe D. J. (2002). J. Microelectromech. Syst..

[cit144] Eddington D. T., Beebe D. J. (2004). J. Microelectromech. Syst..

[cit145] Baldi A., Gu Y., Loftness P. E., Siegel R. A., Ziaie B. (2003). J. Microelectromech. Syst..

[cit146] Kim D., Beebe D. J. (2007). Lab Chip.

[cit147] Kim D., Beebe D. J. (2007). Sens. Actuators, A.

[cit148] Wang Y., Toyoda K., Uesugi K., Morishima K. (2020). Sens. Actuators, A.

[cit149] Zhang J., Du P., Xu D., Li Y., Peng W., Zhang G., Zhang F., Fan X. (2016). Ind. Eng. Chem. Res..

[cit150] Jadhav A. D., Yan B., Luo R. C., Wei L., Zhen X., Chen C. H., Shi P. (2015). Biomicrofluidics.

[cit151] Coleman S., ter Schiphorst J., Azouz A., Ben Bakker S., Schenning A. P. H. J., Diamond D. (2017). Sens. Actuators, B.

[cit152] Delaney C., McCluskey P., Coleman S., Whyte J., Kent N., Diamond D. (2017). Lab Chip.

[cit153] Pandurangan K., Barrett R., Diamond D., McCaul M. (2021). Front. Mater..

[cit154] Sugiura S., Sumaru K., Ohi K., Hiroki K., Takagi T., Kanamori T. (2007). Sens. Actuators, A.

[cit155] Ter Schiphorst J., Coleman S., Stumpel J. E., Ben Azouz A., Diamond D., Schenning A. P. H. J. (2015). Chem. Mater..

[cit156] Cheng Y., Ren K., Huang C., Wei J. (2019). Sens. Actuators, B.

[cit157] Satarkar N. S., Zhang W., Eitel R. E., Hilt J. Z. (2009). Lab Chip.

[cit158] ter Schiphorst J., Melpignano G. G., Amirabadi H. E., Houben M. H. J. M., Bakker S., den Toonder J. M. J., Schenning A. P. H. J. (2018). Macromol. Rapid Commun..

[cit159] Jiao C., Obst F., Geisler M., Che Y., Richter A., Appelhans D., Gaitzsch J., Voit B. (2022). Polymers.

[cit160] Koike Y., Yokoyama Y., Hayakawa T. (2020). Front. Mech. Eng..

[cit161] Prettyman J. B., Eddington D. T. (2011). Sens. Actuators, B.

[cit162] Xiong Z., Dong X. Z., Chen W. Q., Duan X. M. (2008). Appl. Phys. A: Mater. Sci. Process..

[cit163] Richter A., Klatt S., Paschew G., Klenke C. (2009). Lab Chip.

[cit164] Braschler T., Johann R., Heule M., Metref L., Renaud P. (2005). Lab Chip.

[cit165] Ha J. H., Shin H. H., Choi H. W., Lim J. H., Mo S. J., Ahrberg C. D., Lee J. M., Chung B. G. (2020). Lab Chip.

[cit166] Gayet R. V., de Puig H., English M. A., Soenksen L. R., Nguyen P. Q., Mao A. S., Angenent-Mari N. M., Collins J. J. (2020). Nat. Protoc..

[cit167] Ambrožič R., Plazl I. (2021). Soft Matter.

[cit168] Fu F., Shang L., Chen Z., Yu Y., Zhao Y. (2018). Sci. Robot..

[cit169] Sun L., Wang Y., Bian F., Xu D., Zhao Y. (2023). Sci. Bull..

[cit170] Kwon G. H., Choi Y. Y., Park J. Y., Woo D. H., Lee K. B., Kim J. H., Lee S. H. (2010). Lab Chip.

[cit171] Beck A., Mehner P. J., Voigt A., Obst F., Marschner U., Richter A. (2022). Adv. Mater. Technol..

[cit172] Park S., Choi G., Kang M., Kim W., Kim J., Jeong H. E. (2023). Microsyst. Nanoeng..

[cit173] Li J., Su K., Liu H., Zou Y. (2024). Magnetochemistry.

[cit174] Vasić K., Knez Ž., Leitgeb M. (2024). J. Funct. Biomater..

[cit175] Kudr J., Haddad Y., Richtera L., Heger Z., Cernak M., Adam V., Zitka O. (2017). Nanomaterials.

[cit176] Merazzo K. J., Lima A. C., Rincón-Iglesias M., Fernandes L. C., Pereira N., Lanceros-Mendez S., Martins P. (2021). Mater. Horiz..

[cit177] Roshan U., Mudugamuwa A., Cha H., Hettiarachchi S., Zhang J., Nguyen N.-T. (2024). Lab Chip.

[cit178] Tang W., Jiang D., Li Z., Zhu L., Shi J., Yang J., Xiang N. (2019). Electrophoresis.

[cit179] Grützkau A., Radbruch A. (2010). Cytometry, Part A.

[cit180] Doganay S., Cetin L., Ezan M. A., Turgut A. (2020). J. Micromech. Microeng..

[cit181] Mirkhani N., Christiansen M. G., Schuerle S. (2020). Adv. Funct. Mater..

[cit182] Peng X. Y., Peng L., Guo Y. (2021). Adv. Mater. Technol..

[cit183] Sohn S., Lee H., Kee H., Park S. (2023). Adv. Intell. Syst..

[cit184] Wang Y., Li Z., Li D., Chen F., Zhao Q., Qing J., Li X., Yang C., He X., Zhao Y. (2024). Actuators.

[cit185] Veloso-Fernández A., Muñana-González S., Laza J. M., Aguilera-Grande A., Jaramillo D. S., Ruiz-Rubio L., Pérez-Alvaréz L., Vilas-Vilela J. L., Lopes A. C. (2024). React. Funct. Polym..

[cit186] Zhao X., Yao H., Lv Y., Chen Z., Dong L., Huang J., Mi S. (2024). Small.

[cit187] Li X., Chen T., Zheng Z., Gao J., Wu Y., Wu X., Jiang T., Zhu Z., Xu R. X. (2024). Small.

[cit188] Lin G., Liu Y., Huang G., Chen Y., Makarov D., Lin J., Quan Z., Jin D. (2021). Adv. Intell. Syst..

[cit189] Sun J., Shi Z., Li M., Sha J., Zhong M., Chen S., Liu X., Jia S. (2022). J. Magn. Magn. Mater..

[cit190] Feng S., Pan C., Ye H., Liu W., Yang W., Lv Y., Tao S. (2023). Small.

[cit191] Broeren S., Pereira I. F., Wang T., den Toonder J., Wang Y. (2023). Lab Chip.

[cit192] Hajihadi Naghash T., Haghgoo A. M., Bijarchi M. A., Ghassemi M., Shafii M. B. (2024). Sens. Actuators, B.

[cit193] Demirörs A. F., Aykut S., Ganzeboom S., Meier Y. A., Poloni E. (2021). Proc. Natl. Acad. Sci. U. S. A..

[cit194] Li M., Zhang T., Zhang X., Mu J., Zhang W. (2022). Front. Bioeng. Biotechnol..

[cit195] Peng Y., Li C., Jiao Y., Zhu S., Hu Y., Xiong W., Cao Y., Li J., Wu D. (2023). Langmuir.

[cit196] He Y., Yin K., Wang L., Wu T., Deng Q., Dou Y., Arnusch C. J. (2023). Nano Lett..

[cit197] Kichatov B., Korshunov A., Sudakov V., Golubkov A., Ryapolov P. (2024). Colloids Surf., A.

[cit198] Yang M., Wu X., Li H., Cui G., Bai Z., Wang L., Kraft M., Liu G., Wen L. (2021). J. Micromech. Microeng..

[cit199] Phiphattanaphiphop C., Leksakul K., Wanta T., Khamlor T., Phattanakun R. (2022). Micromachines.

[cit200] Wang Z., Wang H., Lin S., Ahmed S., Angers S., Sargent E. H., Kelley S. O. (2022). Nano Lett..

[cit201] Liu J., Lyu X., Zhou Z., Yang L., Zeng J., Yang Y., Zhao Z., Chen R., Tong X., Li J., Liu H., Zou Y. (2023). ACS Appl. Mater. Interfaces.

[cit202] Nian M., Chen B., He M., Hu B. (2024). Anal. Chem..

[cit203] Wang Z., Wang H., Lin S., Angers S., Sargent E. H., Kelley S. O. (2024). Sci. Adv..

[cit204] Xu Y., Huang C., Li D., Liu J., Fu S., Wu X., Xu T. (2023). IEEE Robot. Autom. Lett..

[cit205] Ku H., Wang P., Huang C. (2024). Small.

[cit206] Zhao H., Wen R., Zhang L., Chen L., Li H., Xia F., Song Y. (2024). Adv. Sci..

[cit207] Zhu C., Lu Y., Jiang L., Yu Y. (2021). Adv. Funct. Mater..

[cit208] Dong L., Zhao Y. (2018). Mater. Chem. Front..

[cit209] Rey A. D. (2007). Soft Matter.

[cit210] Nie Z. Z., Wang M., Yang H. (2023). Chem. Eur. J..

[cit211] Shang Y., Wang J., Ikeda T., Jiang L. (2019). J. Mater. Chem. C.

[cit212] Zhang J., Zhang Y., Yang J., Wang X. (2024). Micromachines.

[cit213] Xiao Y. Y., Jiang Z. C., Hou J. B., Chen X. S., Zhao Y. (2022). Soft Matter.

[cit214] Tian X., Guo Y., Zhang J., Ivasishin O. M., Jia J., Yan J. (2024). Small.

[cit215] Zhang L., Liu J., Shang P., Jiang X. F., Hu X., Zhou G. (2024). ACS Appl. Nano Mater..

[cit216] Zhang W., Nan Y., Wu Z., Shen Y., Luo D. (2022). Molecules.

[cit217] Mitchell M. J., Billingsley M. M., Haley R. M., Wechsler M. E., Peppas N. A., Langer R. (2021). Nat. Rev. Drug Discovery.

[cit218] Patra J. K., Das G., Fraceto L. F., Campos E. V. R., Rodriguez-Torres M. del P., Acosta-Torres L. S., Diaz-Torres L. A., Grillo R., Swamy M. K., Sharma S., Habtemariam S., Shin H.-S. (2018). J. Nanobiotechnol..

[cit219] Mehta M., Bui T. A., Yang X., Aksoy Y., Goldys E. M., Deng W. (2023). ACS Mater. Au.

[cit220] Gaviria-Arroyave M. I., Cano J. B., Peñuela G. A. (2020). Talanta Open.

[cit221] Lohcharoenkal W., Abbas Z., Rojanasakul Y. (2021). Biosensors.

[cit222] Ahn J., Ko J., Lee S., Yu J., Kim Y., Jeon N. L. (2018). Adv. Drug Delivery Rev..

[cit223] Gimondi S., Ferreira H., Reis R. L., Neves N. M. (2023). ACS Nano.

[cit224] Bezelya A., Küçüktürkmen B., Bozkır A. (2023). Micro.

[cit225] Hang Y., Wang A., Wu N. (2024). Chem. Soc. Rev..

[cit226] Liu Y., Zhang X. (2021). Micromachines.

[cit227] Li Y., Verbiest T., Strobbe R., Vankelecom I. F. J. (2014). J. Mater. Chem. A.

[cit228] Govorov A. O., Zhang W., Skeini T., Richardson H., Lee J., Kotov N. A. (2006). Nanoscale Res. Lett..

[cit229] Povolotskiy A. V., Smirnova O. S., Soldatova D. A., Povolotckaia A. V., Lukyanov D. A. (2023). Metals.

[cit230] Schlicke H., Battista D., Kunze S., Schröter C. J., Eich M., Vossmeyer T. (2015). ACS Appl. Mater. Interfaces.

[cit231] Lucas T. M., Moiseeva E. V., Zhang G., Gobin A. M., Harnett C. K. (2013). Sens. Actuators, A.

[cit232] Reismann M., Bretschneider J. C., von Plessen G., Simon U. (2008). Small.

[cit233] Li Z., Wang P., Tong L., Zhang L. (2013). Opt. Express.

[cit234] Schepperle M., Ghanam M., Bucherer A., Gerach T., Woias P. (2022). Sens. Actuators, A.

[cit235] Liu X., Wang Y., Peng Y., Shi J., Chen W., Wang W., Ma X. (2023). ACS Nano.

[cit236] Arqué X., Patiño T., Sánchez S. (2022). Chem. Sci..

[cit237] Yuan H., Liu X., Wang L., Ma X. (2021). Bioact. Mater..

[cit238] Yang Q., Gao Y., Xu L., Hong W., She Y., Yang G. (2021). Int. J. Biol. Macromol..

[cit239] Choi H., Jeong S. H., Kim T. Y., Yi J., Hahn S. K. (2022). Bioact. Mater..

[cit240] Draz M. S., Kochehbyoki K. M., Vasan A., Battalapalli D., Sreeram A., Kanakasabapathy M. K., Kallakuri S., Tsibris A., Kuritzkes D. R., Shafiee H. (2018). Nat. Commun..

[cit241] Qin F., Wu J., Fu D., Feng Y., Gao C., Xie D., Fu S., Liu S., Wilson D. A., Peng F. (2022). Appl. Mater. Today.

[cit242] Patino T., Porchetta A., Jannasch A., Lladó A., Stumpp T., Schäffer E., Ricci F., Sánchez S. (2019). Nano Lett..

[cit243] Vizsnyiczai G., Frangipane G., Maggi C., Saglimbeni F., Bianchi S., Di Leonardo R. (2017). Nat. Commun..

[cit244] Cuntín-Abal C., Bujalance-Fernández J., Yuan K., Arribi A., Jurado-Sánchez B., Escarpa A. (2024). Adv. Funct. Mater..

[cit245] Donato S., Nocentini S., Martella D., Kolagatla S., Wiersma D. S., Parmeggiani C., Delaney C., Florea L. (2024). Small.

[cit246] Ennis A., Nicdao D., Kolagatla S., Dowling L., Tskhe Y., Thompson A. J., Trimble D., Delaney C., Florea L. (2023). Adv. Funct. Mater..

[cit247] Augustine A., Qian J., Faraone T., Kolagatla S., Prochukhan N., Morris M. A., Bradley A. L., Florea L., Delaney C. (2024). Small.

[cit248] Delaney C., Qian J., Zhang X., Potyrailo R., Bradley A. L., Florea L. (2021). J. Mater. Chem. C.

[cit249] Wang L., Sai H., Tang Y., Li B., Wang L., Yang Y., Yang K., Lv P., Duan H., Huang T. (2025). Adv. Funct. Mater..

[cit250] Hsu L. Y., Mainik P., Münchinger A., Lindenthal S., Spratte T., Welle A., Zaumseil J., Selhuber-Unkel C., Wegener M., Blasco E. (2023). Adv. Mater. Technol..

[cit251] Ennis A., Nicdao D., Kolagatla S., Dowling L., Tskhe Y., Thompson A. J., Trimble D., Delaney C., Florea L. (2023). Adv. Funct. Mater..

[cit252] Spiegel C. A., Hackner M., Bothe V. P., Spatz J. P., Blasco E. (2022). Adv. Funct. Mater..

[cit253] Elliott L. V., Salzman E. E., Greer J. R. (2021). Adv. Funct. Mater..

[cit254] del Pozo M., Delaney C., Pilz da Cunha M., Debije M. G., Florea L., Schenning A. P. H. J. (2022). Small Struct..

[cit255] Hippler M., Blasco E., Qu J., Tanaka M., Barner-Kowollik C., Wegener M., Bastmeyer M. (2019). Nat. Commun..

[cit256] Spratte T., Geiger S., Colombo F., Mishra A., Taale M., Hsu L. Y., Blasco E., Selhuber-Unkel C. (2023). Adv. Mater. Technol..

[cit257] Saetchnikov A. V., Tcherniavskaia E. A., Saetchnikov V. A., Ostendorf A. (2024). Light: Adv. Manuf..

[cit258] Del Pozo M., Delaney C., Bastiaansen C. W. M., Diamond D., Schenning A. P. H. J., Florea L. (2020). ACS Nano.

[cit259] Qian J., Kolagatla S., Pacalovas A., Zhang X., Florea L., Bradley A. L., Delaney C. (2023). Adv. Funct. Mater..

[cit260] Delaney C., Qian J., Zhang X., Potyrailo R., Bradley A. L., Florea L. (2021). J. Mater. Chem. C.

[cit261] Zhang W., Wang H., Wang H., Chan J. Y. E., Liu H., Zhang B., Zhang Y. F., Agarwal K., Yang X., Ranganath A. S., Low H. Y., Ge Q., Yang J. K. W. (2021). Nat. Commun..

[cit262] Barwig C., Sonn A., Spratte T., Mishra A., Blasco E., Selhuber-Unkel C., Pashapour S. (2024). Adv. Intell. Syst..

[cit263] Tudor A., Delaney C., Zhang H., Thompson A. J., Curto V. F., Yang G. Z., Higgins M. J., Diamond D., Florea L. (2018). Mater. Today.

[cit264] Hu Y., Wang Z., Jin D., Zhang C., Sun R., Li Z., Hu K., Ni J., Cai Z., Pan D., Wang X., Zhu W., Li J., Wu D., Zhang L., Chu J. (2020). Adv. Funct. Mater..

